# Associations between transport modes and site-specific cancers: a systematic review and meta-analysis

**DOI:** 10.1186/s12940-024-01081-3

**Published:** 2024-04-13

**Authors:** Win Thu, Alistair Woodward, Alana Cavadino, Sandar Tin Tin

**Affiliations:** 1 Epidemiology and Biostatistics, School of Population Health, University of Auckland, Auckland, New Zealand; 2 Cancer Epidemiology Unit, Oxford Population Health, University of Oxford, Oxford, UK

**Keywords:** Transport modes, Active transport, Site-specific cancers, Systematic review, Meta-analysis

## Abstract

**Background:**

Physical inactivity is a global public health problem. A practical solution would be to build physical activity into the daily routine by using active modes of transport. Choice of transport mode can influence cancer risk through their effects on levels of physical activity, sedentary time, and environmental pollution. This review synthesizes existing evidence on the associations of specific transport modes with risks of site-specific cancers.

**Methods:**

Relevant literature was searched in PubMed, Embase, and Scopus from 1914 to 17th February 2023. For cancer sites with effect measures available for a specific transport mode from two or more studies, random effects meta-analyses were performed to pool relative risks (RR) comparing the highest vs. lowest activity group as well as per 10 Metabolic Equivalent of Task (MET) hour increment in transport-related physical activity per week (∼150 min of walking or 90 min of cycling).

**Results:**

27 eligible studies (11 cohort, 15 case-control, and 1 case-cohort) were identified, which reported the associations of transport modes with 10 site-specific cancers. In the meta-analysis, 10 MET hour increment in transport-related physical activity per week was associated with a reduction in risk for endometrial cancer (RR: 0.91, 95% CI: 0.83–0.997), colorectal cancer (RR: 0.95, 95% CI: 0.91–0.99) and breast cancer (RR: 0.99, 95% CI: 0.89–0.996). The highest level of walking only or walking and cycling combined modes, compared to the lowest level, were significantly associated with a 12% and 30% reduced risk of breast and endometrial cancers respectively. Cycling, compared to motorized modes, was associated with a lower risk of overall cancer incidence and mortality.

**Conclusion:**

Active transport appears to reduce cancer risk, but evidence for cancer sites other than colorectum, breast, and endometrium is currently limited.

**Supplementary Information:**

The online version contains supplementary material available at 10.1186/s12940-024-01081-3.

## Introduction

Physical inactivity is a global public health problem, contributing to substantial disease and economic burden worldwide [1, 2]. With rapid changes in technology, lifestyle, and habitual environment, people have been less active and more sedentary over the past few decades. Globally, about 1 in 4 adults were not active, i.e., did not meet the World Health Organization (WHO) recommendation of engaging at least 150–300 min of moderate-intensity or 75–150 min of vigorous-intensity aerobic physical activity per week [3], but the prevalence varied widely within and across countries [4]. If the current trends continue, it is unlikely that the WHO’s target to reduce physical inactivity by 10% in 2025 will be met.

One practical solution would be to build physical activity into the daily routine by using active modes of transport [5]. Walking and cycling have been shown to improve health (mainly all-cause mortality, cardiovascular disease, diabetes, and cancer) [6] and also provide social, economic and environmental benefits [7, 8]. Car use, on the other hand, contributes to a significant proportion of daily sedentary time, and the situation is worsening with increasing traffic congestion/delays [9]; it has been associated with an increased risk of obesity and related outcomes [10]. Further, exposure to environmental pollutants such as nitrogen dioxide and/or particulate matter could differ across different road users [11], while it has been shown to increase the risk of certain cancers, particularly lung cancer [12].

While there is ample evidence linking leisure time physical activity or physical activity in general with a reduced risk for a number of cancer sites [13, 14], and sedentary behavior in general with an increased risk [15], the findings may not be directly applicable to transport-related activity because the context and correlates of activity as well as its frequency, duration and intensity are likely to be different across different domains. We therefore reviewed the existing literature that reported the associations between transport modes and risks of site-specific cancers.

## Methods

A systematic literature review and meta-analysis was conducted and reported according to the PRISMA guideline (Supplementary file S1). The review was not registered.

### Search strategy and study selection

Relevant literature was searched from 1914 to 17th February 2023 in PubMed, Scopus, and Embase databases using the relevant search terms such as walking, cycling, car, public transport, commute and cancers. Site-specific cancers known to be associated with physical activity and body weight such as breast, colon, liver, esophageal adenocarcinoma and those associated with environmental factor such as lung and melanoma of skin were also searched (Supplementary file S2). The reference lists of systematic reviews on physical activity and cancers were also reviewed. Studies were included if they (1) used cohort, case-control, case-cohort or experimental design, (2) assessed transport modes such as walking, cycling, public transport or car use as the exposures of interest, (3) investigated one or more site-specific cancers, overall cancer incidence and/or mortality as the outcome(s), (4) reported effect measures associated with transport modes, and (5) published the full article in English. Studies that used cross-sectional design or mathematical modeling to estimate health impacts at the population level were excluded. Details of excluded studies after full text review, together with the reasons for exclusion, were provided in the Supplementary file S3. WT conducted the search and selection, and STT oversaw the process.

### Data extraction and study quality assessment

Information about title, first author, year of publication, study name (if available), country, study design, sample size, age range of the participants, follow-up duration (for cohort and case-cohort studies), data collection tool, measurement units for exposure(s), data sources for outcome(s), site-specific cancer assessed, effect measures, and confounders adjusted were extracted in a standardized data collection spreadsheet. The study quality was evaluated using the Newcastle-Ottawa Scale (NOS) [16], which scores the cohort and case-control studies based on three domains: selection of study groups, comparability of the groups and ascertainment of exposure (case-control studies) or outcome (cohort studies). For the second domain, a point was awarded for adjustment of Body Mass Index (BMI) - to evaluate the direct vs. indirect (through BMI) effect of physical activity on cancer risk, and another point for adjustment of physical activities from other domains - to isolate the effects of transport-related physical activity from other activities. A maximum of nine points were awarded, with a higher score indicating better quality [16]. For case-cohort studies, the NOS scale for cohort studies was used. WT conducted the data extraction and quality assessment, and STT oversaw the process.

### Data analysis

For cancer sites with effect measures available for a specific transport mode from two or more studies, meta-analyses were performed using random effects models. The analyses compared the highest level of active transport such as walking, cycling or mixed mode with the lowest level as reported in the individual studies. Where necessary, the reference category for exposure was changed to the lowest group to facilitate pooling of the risks [17]. The pooled relative risks (RRs) and 95% CI were presented for breast, endometrial, colorectal and testicular cancers, and overall cancer mortality.

For studies that reported time or MET as measurement units, the dose-response effects were estimated using the trend estimation method proposed by Greenland and Longnecker [18]. The reported time spent for each mode/category was converted to MET hours (see Supplementary file S4 for conversion values and formulas used). For studies that only reported estimates for categorical exposures, study-specific slopes were calculated from the natural logs of the reported risk estimates across categories and risk estimates per unit change were then estimated. The pooled results were presented per 10 Metabolic Equivalent of Task (MET) hour increment in transport-related physical activity per week (∼150 min of walking or 90 min of cycling) to align with the WHO’s physical activity recommendation [3]. This approach enabled us to pool risk estimates from a large number of studies irrespective of how the exposures were assessed (e.g., walking and cycling separately or combined) or categorised. The results were presented for breast, endometrial, colorectal, prostate cancers, and overall cancer mortality.

Meta-analysis was not conducted for the studies that compared active and non-active modes in relation to overall cancer incidence and mortality due to the potential overlap of the study samples.

For meta-analyses involving four or more studies, publication bias was assessed through the visual inspection of funnel plots, Begg’s rank correlation test, and Egger’s regression test for asymmetry. If significant associations were observed, sensitivity analyses were conducted by removing one study at a time from the initial meta-analysis to test the robustness of the results. Where possible, sub-group analyses were performed to assess variability of summary effects across population groups (Western vs. Asian), study design (cohort vs. case-control), measurement units (time vs. MET) and adjustment for BMI (yes vs. no). Metafor [19] and dosresmeta [20] R packages were used for meta-analysis and trend estimation. All authors have access to the data.

## Results

**Fig. 1:**
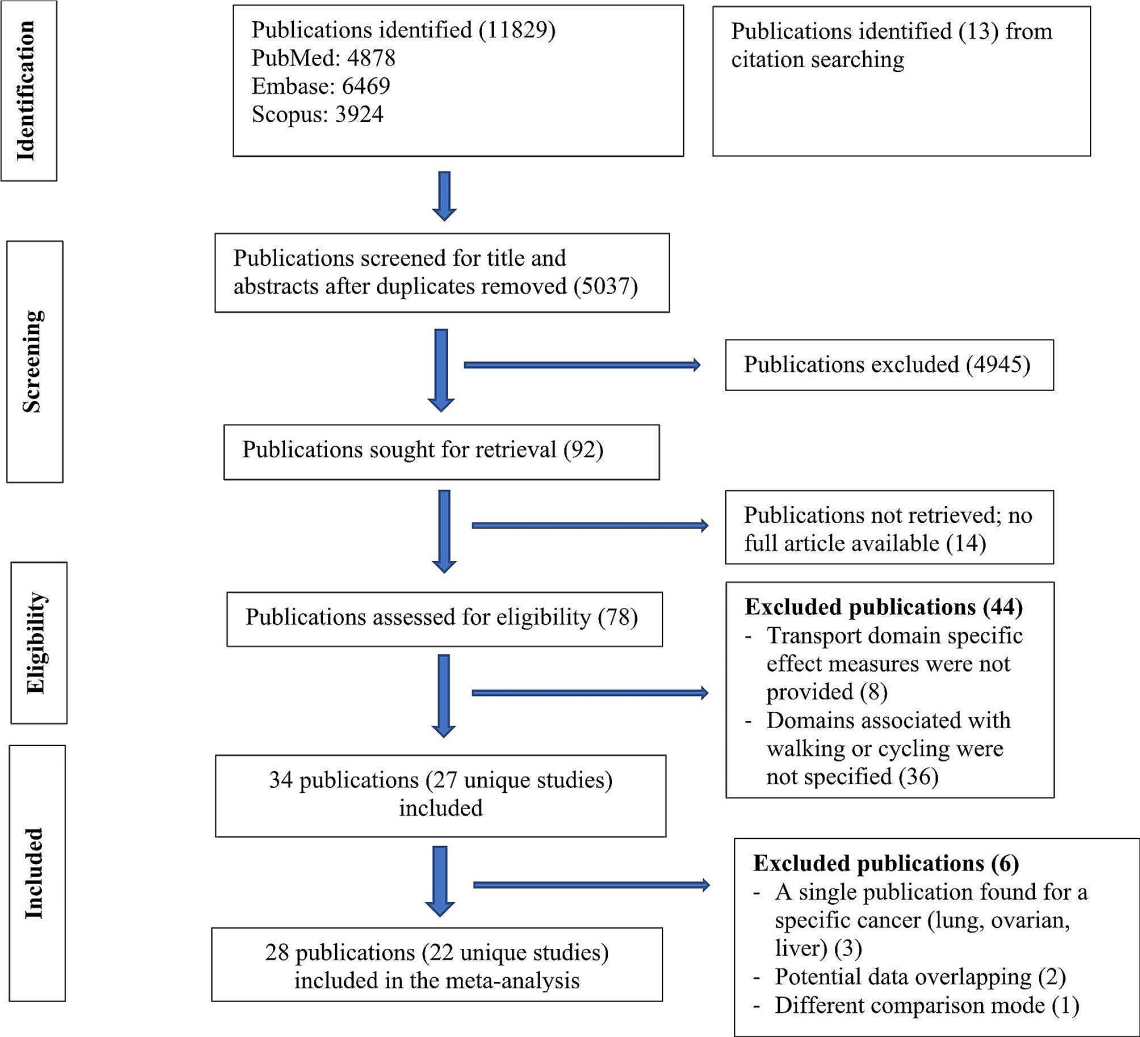
Flow diagram for study selection

Of the 11,829 records identified, 27 unique studies (total 34 publications) were included, of which 22 studies (28 records) contributed to the meta-analyses (Fig. 1). There were four publications from the Netherlands Cohort Study which reported endometrial [21], ovarian [22], prostate [23], and colorectal [24] cancers, three publications from United Kingdom Biobank which reported lung [25], breast and colon [26], and overall cancer incidence and mortality [27], two publications from Shanghai Women’s Health Study which reported breast [28] and overall cancer mortality [29], and two publications from National Institutes of Health - American Association of Retired Persons Diet and Health Study which reported breast [30] and endometrial [31] cancers. Of the included studies, 20 compared the risks between the highest and lowest levels of active transport (e.g., walking, cycling, walking and cycling) and two compared the risk between active and non-active commuting modes. The majority used case-control design (*n* = 15), followed by cohort (*n* = 11) and case-cohort (*n* = 1) designs. Most of the studies were conducted in North America, mainly in the United States (US) (*n* = 7), followed by Europe (*n* = 5), China (*n* = 5), United Kingdom (*n* = 4), Australia (*n* = 2) and the remaining four studies were from India, Iran, Brazil and Nigeria. (Table 1)

**Fig. 2:**
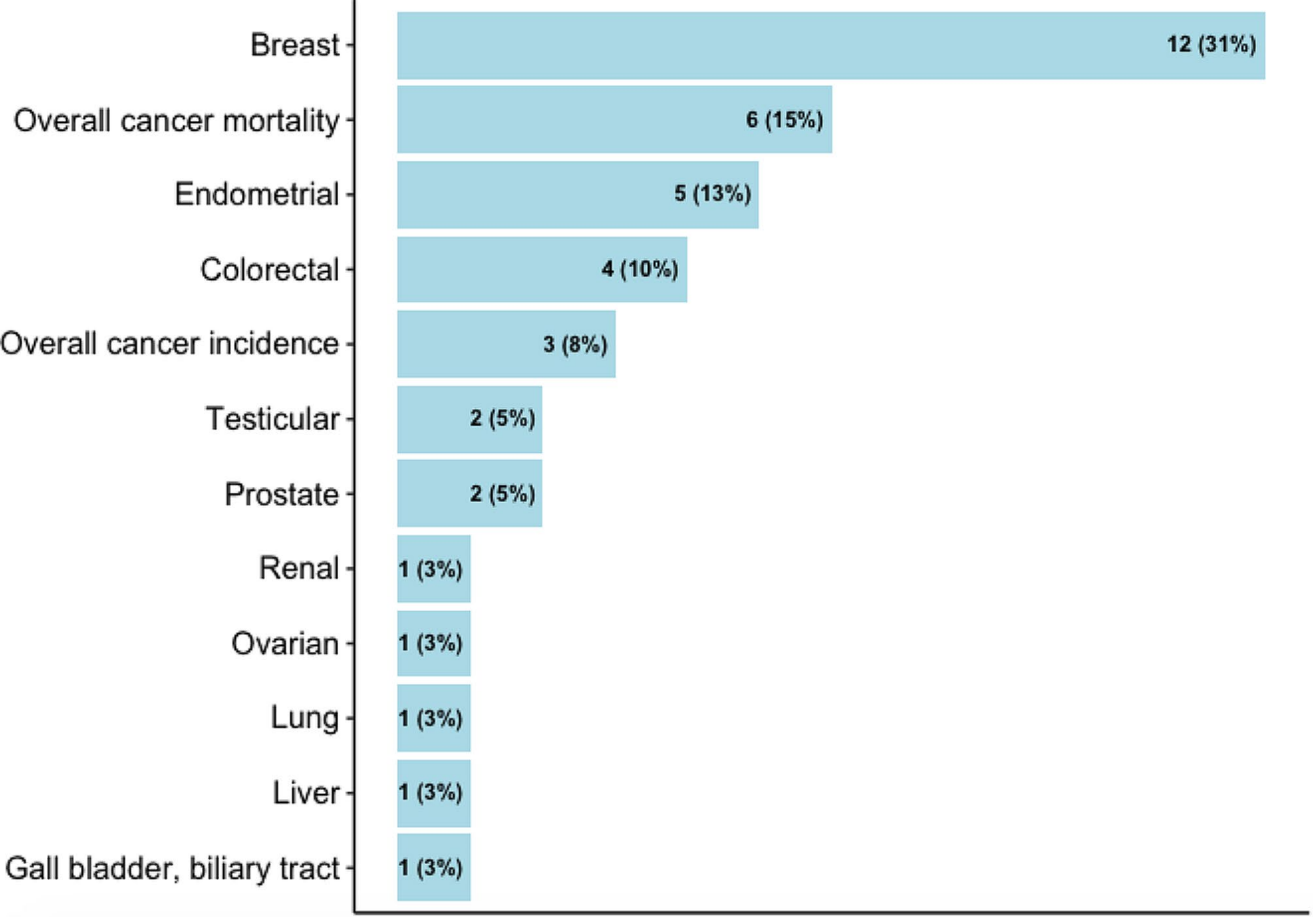
Cancers reported in the studies

Almost half of the studies assessed walking and cycling combined, i.e., did not provide the risk estimates for each mode (*n* = 13), while others assessed walking and cycling separately (*n* = 8), or assessed only one mode (walking: *n* = 3 and cycling: *n* = 3). Most studies quantified active transport in terms of time spent (e.g., minutes per day, hours per week) (*n* = 14) or MET (*n* = 7), but others assessed it in terms of activity status (e.g., yes, no) (*n* = 3), or in comparison to car or motorized mode (*n* = 2), and distance (*n* = 1) (Supplementary file S5). The studies reported the risks associated with ten site-specific cancers, most commonly breast (*n* = 12), endometrial (*n* = 5), and colorectal (*n* = 4) cancers (Fig. 2). Cancer cases were identified through cancer registries, death registries, pathological reports, or hospital or medical records (Supplementary file S5). The NOS score for cohort studies ranged from 5 to 9, with an average score of 6.5, and the score for case-control studies ranged from 4 to 7, with an average score of 5.6 (Table 1, detailed scoring in Supplementary file S6, S7).

**Table 1:** Study design, outcomes and modes assessed in the studies

Author, year	Study design, country	Participants, cases^*^, follow up years	Assessed modes and reference^†^	NOS score^‡^
**Breast cancer**				
Panter et al. [26]	Cohort, UK	358,799 participants, 1,139 cases, 7 years	Active patterns of commuting^§^ (reference mode: car only mode)	6
Pronk et al. [28]	Cohort, China	73,049 participants, 717 cases, 9 years	Walking and cycling separately	7
George et al. [30]	Cohort, USA	97,039 participants, 2,866 cases Postmenopausal, 7 years	Walking and cycling combined	6
Luoto et al. [32]	Cohort, Finland	30,548 participants, 332 cases 6 years	Walking and cycling combined	5
Gomes et al. [33]	Case-control, Brazil	231 controls, 230 cases	Walking and cycling combined	5
Azubuike et al. [34]	Case-control, Nigeria	403 controls, 288 cases	Walking only	5
Si et al. [35]	Case-control, Australia	1,789 controls, 1,205 cases, Premenopausal, Postmenopausal	Walking and cycling combined	6
Mathew et al. [36]	Case-control, India	1,873 controls, 1,866 cases Premenopausal, Postmenopausal	Walking only	6
Steindorf et al. [37]	Case-control, Germany	886 controls, 359 cases	Walking and cycling separately	6
John et al. [38]	Case-control, US	1,548 controls, 1,250 cases Premenopausal, Postmenopausal	Walking and cycling combined	7
Matthews et al. [39]	Case-control, China	1,556 controls, 1,459 cases	Walking and cycling separately	5
Marcus et al. [40]	Case-control, US	790 controls, 861 cases	Walking and cycling separately	5
**Endometrial**				
Gierach et al. [31]	Cohort, US	109,621 participants, 647 cases, 7 years	Walking and cycling combined	7
Friberg et al. [41]	Cohort, Sweden	33,723 participants, 199 cases, 18 years	Walking and cycling combined	6
Schouten et al. [21]	Case-cohort The Netherlands	62,573 participants, 226 cases, 9 years	Walking and cycling combined	6
John et al. [42]	Case-control, US	443 controls, 472 cases	Walking and cycling combined	6
Matthews et al. [43]	Case-control, China	846 controls, 832 cases	Walking and cycling separately	7
**Colorectal cancer**				
Mahmood et al. [44]	Cohort, Australia	23,586 participants, 465 cases Colorectal, 16-years	Walking and cycling combined	6
Panter et al. [26]	Cohort, UK	358,799 participants, 435 cases Colon, 7 years	Active patterns of commuting^§^ (reference mode: car only mode)	6
Simons et al. [24]	Case-cohort, The Netherlands	120,852 participants, 3,185 cases, Colon, rectum, 16 years	Walking and cycling combined	7
Hou et al. [45]	Case-control, China	1,552 controls, 931 cases Colon	Walking and cycling separately	6
**Testicular**				
Littman et al. [46]	Case-control, US	1023 controls, 391 cases	Cycling only	6
Coldman et al. [47]	Case-control, US	128 controls, 40 cases	Cycling only	6
**Prostate**				
Zeegers et al. [23]	Case-cohort, The Netherlands	58,279 participants, 1,352 cases 9.3 years	Walking and cycling combined	7
Hosseini et al. [48]	Case-control, Iran	137 controls, 137 cases	Walking only	4
**Ovarian**				
Biesma et al. [22]	Case-cohort, The Netherlands	62,573 participants, 252 cases 11.3 years	Walking and cycling combined	7
**Liver, gallbladder and biliary tract**				
Pang et al. [49]	Cohort, China	460,937 participants, 13 years Liver, gallbladder and biliary tract	Commuting physical activity	7
**Renal cancer**				
Xiao et al. [50]	Case-control, US, Renal cell carcinoma	1235 controls, 1217 cases	Walking and cycling combined	5
**Lung**				
Wong et al. [25]	Cohort, UK	234,124 participants, 493 cases 7 years	Walking, cycling, public transport (reference mode: automobile only)	6
**Overall cancer incidence**				
Patterson et al. [51]	Cohort, UK	394,746 participants, 20,980 cases, 16 years	Walking, cycling, public transport (reference mode: car/motorcycle)	6
Panter et al. [26]	Cohort, UK	358,799 participants, 6,216 cases 7 years	Active patterns of commuting^§^ (reference mode: car only mode)	6
Morales et al. [27]	Cohort, UK	263,540 participants, 3748 cases 2 years	Walking, cycling, mixed mode (walking), mixed mode (cycling) (reference mode: non-active mode - car/public transport)	5
**Overall cancer mortality**				
Patterson et al. [51]	Cohort, UK	394,746 participants, 6509 cases 16 years	Walking, cycling, public transport (reference mode: car/motorcycle)	6
Panter et al. [26]	Cohort, UK	358,799 participants, 737 cases 7 years	Active patterns of commuting^§^ (reference mode: car only mode)	6
Morales et al. [27]	Cohort, UK	263,540 participants, 1,123 cases, 2 years	Walking, cycling, mixed mode (walking), mixed mode (cycling) (reference mode: non-active mode - car/public transport)	5
Sahlqvist et al. [52]	Cohort, Europe	13,346 participants, 700 cases 11.5 years	Cycling	6
Autenrieth et al. [53]	Cohort, Germany	4,672 participants, 326 cases, 18 years	Walking and cycling combined	9
Matthews et al. [29]	Cohort, China	67,143 women, 537 cases 5.7 years	Walking and cycling separately	7
Batty et al. [54]	Cohort, UK	11,663 participants, 1,499 cases 25 years	Walking and cycling combined	7

^*^Only number of cases specific for transport mode

^†^Comparison is between the highest and lowest levels of assessed mode/s if not specify with reference mode

^‡^Control of body mass index and physical activities from other domains, and five or more years of follow up in the cohort and case-cohort studies were awarded a point each

^§^Any other patterns including walking, cycling, public transport, either alone or in combination with car, NOS = Newcastle-Ottawa Scale

### Active transport studies

**Fig. 3:**
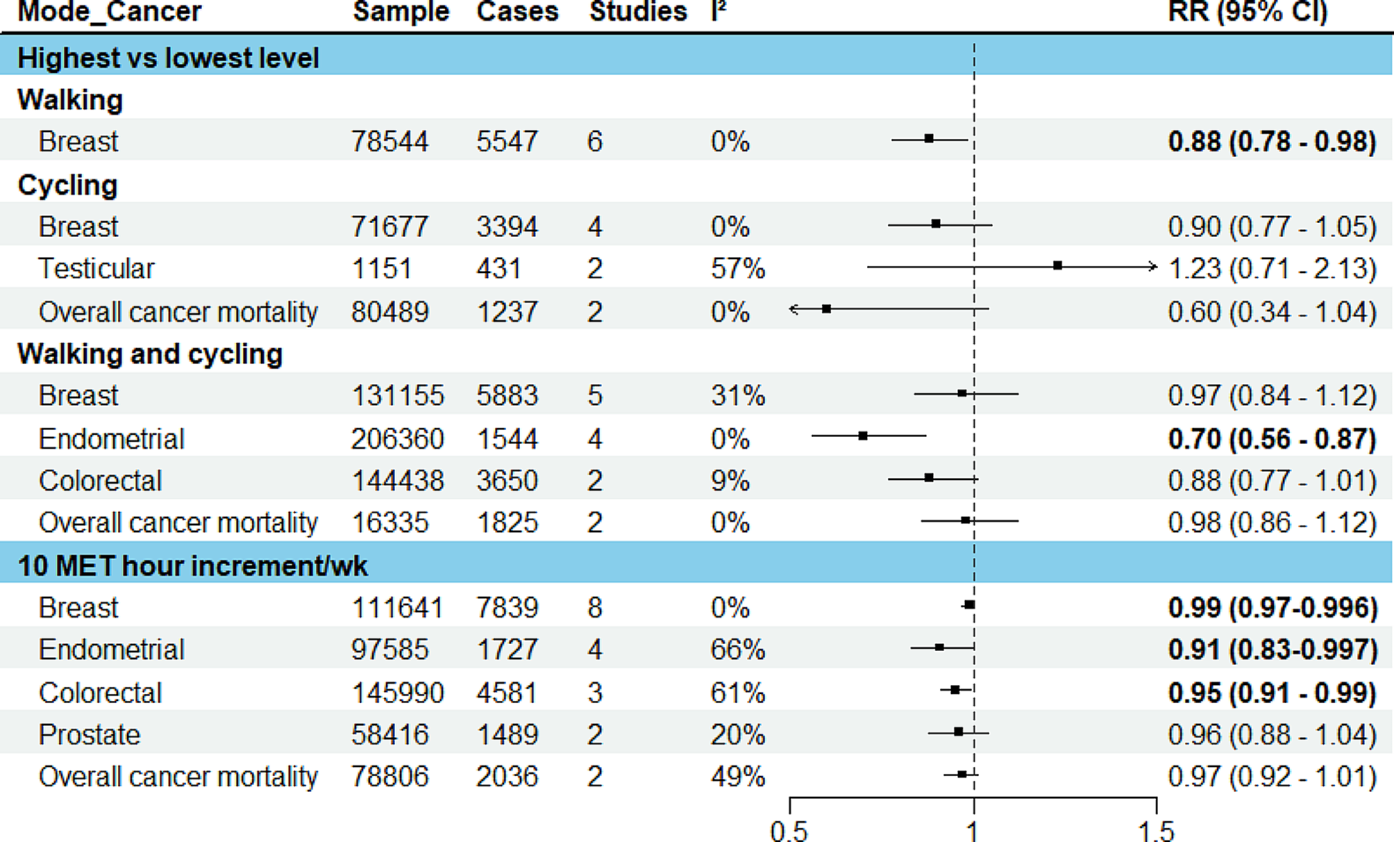
Results of meta-analysis for active transport studies. RE = a random-effects model, MET = Metabolic Equivalent of Task, I^2^ = I^2^ statistics for heterogeneity, RR = Summary relative risk

The pooled results were presented for breast, endometrial, colorectal, testicular and prostate cancers, and overall cancer mortality (Fig. 3). For other cancers where only one study was identified, the results from the individual study were presented.

### Breast cancer

In the meta-analysis of six studies comparing the highest vs. lowest activity group, an inverse association was observed for walking (RR: 0.88, 95% CI: 0.78–0.98), a borderline inverse association for cycling (RR: 0.90, 95% CI: 0.77–1.05) and no significant association for walking and cycling combined (RR: 0.97, 95% CI: 0.84–1.12). 10 MET hour increment in transport-related physical activity per week (∼150 min of walking or 90 min of cycling) was associated with a marginally reduced risk (RR: 0.99, 95% CI: 0.97–0.996). (Fig. 3, detailed forest plots in the supplementary file S9)

### Endometrial cancer

The meta-analysis of four studies indicated that walking and cycling combined was associated with a reduced risk of endometrial cancer (RR comparing highest vs. lowest: 0.70, 95% CI: 0.56–0.87; RR per 10 MET hour increment in activity per week: 0.91, 95% CI: 0.83–0.997). (Fig. 3, detailed forest plots in S9)

### Colorectal cancer

In the meta-analysis of two studies, walking and cycling combined was associated with a reduced risk of colorectal cancer (RR comparing highest vs. lowest: 0.89, 95% CI: 0.78–1.01; RR per 10 MET hour increment in activity per week: 0.95, 95% CI: 0.91–0.99) (Fig. 3, detailed forest plot in S9).

### Testicular cancer

In the meta-analysis of two studies, there was no significant association between cycle commuting in adolescence and testicular germ cell cancer (RR comparing highest vs. lowest: 1.23, 95% CI: 0.71–2.13). (Fig. 3, detailed forest plot in S9)

### Prostate cancer

10 MET hour increment per week for transport related physical activity was associated with a reduced risk of prostate cancer (RR: 0.96, 95% CI: 0.88–1.04) (Fig. 3, detailed forest plot in S9).

### Ovarian cancer

Only a case-cohort study assessed the relationship of walking and cycling combined mode with ovarian cancer risk, and reported no significant association (Supplementary file S5) [22].

### Liver, gallbladder and biliary tract cancers

A cohort study reported a significant association of commuting physical activity with a reduced risk of gallbladder and biliary tract cancers in women (HR: 0.51, 95% CI: 0.28–0.94) but not in men (HR: 0.92, 95% CI: 0.61–1.37); there was no significant association with liver cancer in both sexes (supplementary file S5) [49].

### Renal cancer

A case-control study assessed the association of walking and cycling with risk of renal cell carcinoma in white and black participants in the ages of 20s and 50s, and reported a significant association in the white participants in their 20s (OR comparing lowest vs. highest: 1.42, 95% CI: 1.10–1.83) but not in the black counterparts; the associations were also not significant in both groups in their 50s. (Supplementary file S5) [50].

### Overall cancer mortality

In the meta-analyses of two studies, there was an inverse association for cycling only (RR comparing highest vs. lowest: 0.60, 95% CI: 0.34–1.04) and walking and cycling combined (RR: 0.98, 95% CI: 0.86–1.12), and also per 10 MET hour increment in activity per week (RR: 0.97, 95% CI: 0.92–1.01). (Fig. 3, detailed forest plots in S9)

In sub-group analyses, similar associations were observed between walking and breast cancer risk in terms of study design (cohort, case-control), population (western vs. Asian), measurement unit (time vs. MET), menopausal status (premenopausal and postmenopausal) and adjustment of BMI (yes vs. no); however, the associations were stronger in studies that adjusted for physical activity from other domains (Supplementary file S10). In the leave-one-out analyses assessing walking and breast cancer risk, the results were sensitive to effect sizes from some studies, but this was not the case for walking and cycling combined mode and endometrial cancer (Supplementary file S11). There was no evidence for funnel plot asymmetry; Egger’s regression tests and Begg’s ranks correlation tests were not significant (Supplementary file S12).

### Studies comparing active vs. non-active modes for commuting

**Fig. 4:**
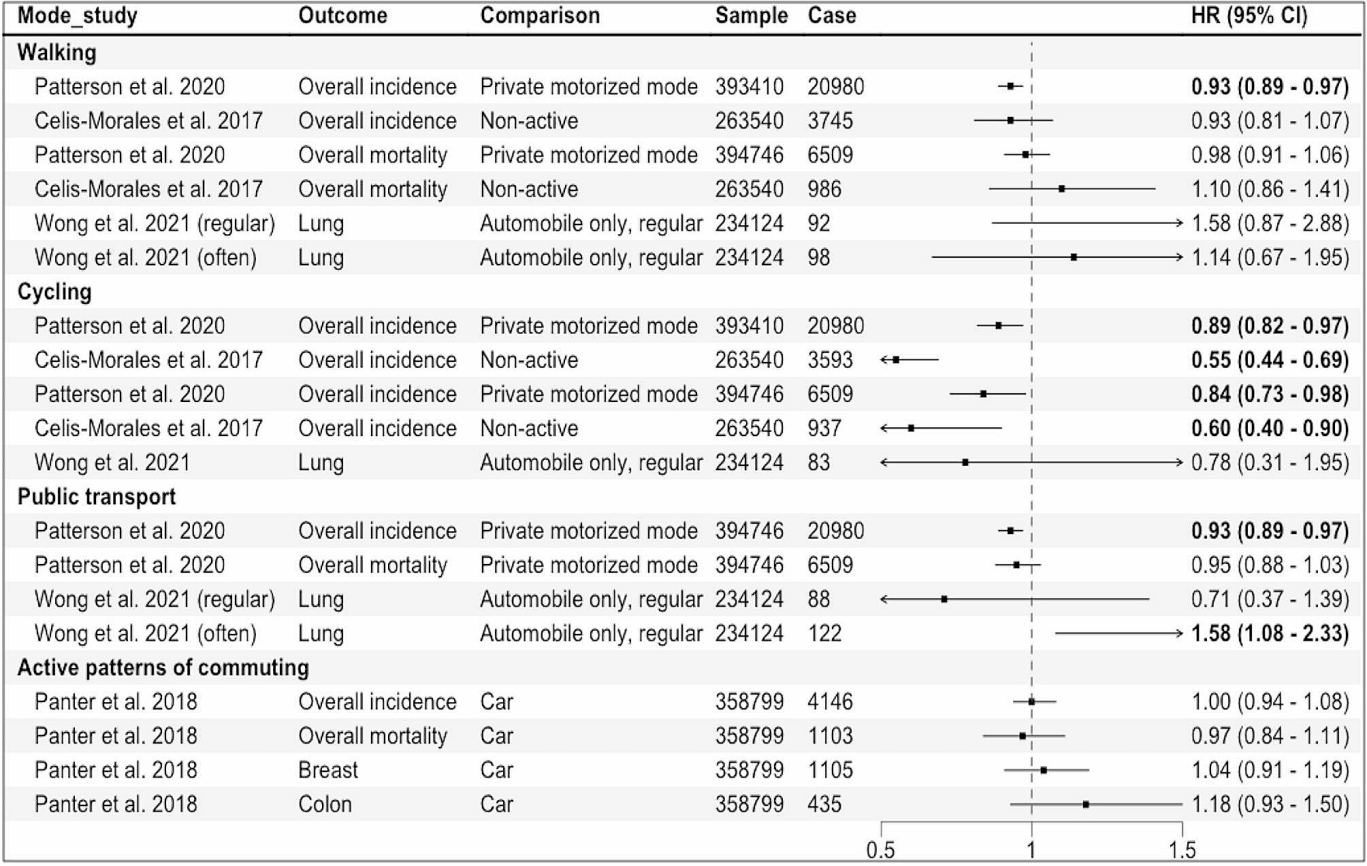
Results of the individual studies comparing active vs. non-active modes for commuting. Private motorized mode = car or motorcycle, Non-active = car or public transport, Active patterns of commuting = any other patterns including walking, cycling, public transport, either alone or in combination with car, HR = Hazard Ratio, regular:1–4, often: ≥5 work-bound trips/week

Four eligible publications were identified, of which three used the data from UK Biobank [25–27], one used the UK census data [51]. Three reported the associations for overall cancer incidence and mortality, and one reported the risk associated with lung cancer (Fig. 4). In the study that assessed lung cancer using the data from UK Biobank, when compared to automobile only mode, active modes did not show a significant association whereas frequent use of public transport (≥ 5 trips per week) was associated with an increased risk of lung cancer (HR: 1.58, 95% CI: 1.08–2.33) [25] (Fig. 4). In another UK Biobank study, no significant associations were observed for breast and colon cancers, and overall cancer incidence and mortality when more active patterns of commuting (walking, cycling, public transport, either alone or in combination with car) were compared to car only mode [26].

The results of two studies [27, 51] that assessed overall cancer incidence and mortality were not combined as the outcome data was extracted from the same national cancer registry with an overlapped time frame (1991–2011 and 2007–2014), although the exposure information came from different sources (census and UK Biobank). In these studies, compared to private motorized mode or non-active mode, cycling was inversely associated with overall cancer incidence and mortality. Walking and public transport were also inversely associated with overall cancer incidence in the study that used the census data [51].

## Discussion

This review identified 27 studies (34 publications) that reported the associations of specific transport modes, mainly active transport modes, with risks of ten site-specific cancers along with overall cancer incidence and mortality. The most frequently studied cancer sites were breast, endometrium, and colorectum; our meta-analysis showed a reduction in risk of these cancers (1%, 9% and 5%, respectively) per 10 MET hour per week increment in transport-related physical activity (∼150 min of walking or 90 min of cycling).

We found an inverse association between active transport and risks of breast and endometrial cancers, with similar magnitude of risk reduction observed in previous systematic reviews on physical activity in general [55, 56]. While obesity is known to increase post-menopausal but not pre-menopausal breast cancer risk [57], we found similar results by menopausal status. In contrast, an earlier review did not find any significant association between walking in general and risk of pre- or post-menopausal breast cancer [58], possibly because compared to walking for transport, walking for leisure or at home generally uses lower energy [59], and therefore may have less effect on body weight.

The inverse association of active transport with colorectal cancer risk observed in this review is also consistent with the findings from existing reviews on transport-related physical activity [60] as well as physical activity in general [61]. While physical activity in general or for leisure has also been associated with a reduced risk of many other cancer sites including liver, gastric, renal and lung [13, 14], the evidence related to transport-related physical activity is currently limited.

Mechanisms linking physical activity with specific cancer sites have been proposed, including its effects on sex hormones (breast, endometrial and prostate cancers), insulin sensitivity, glucose metabolism and adipokines (obesity-related cancers), and inflammation and immune function (most cancers) [62]. For colorectal cancer, another potential mechanism is reduced contact time between carcinogens and bowel mucosa cells due to exercise-induced intestinal mobility [63].

The overall quality of the included studies, evaluated by NOS score, ranged from 4 to 9, and in general, cohort studies tend to have higher scores compared to case-control studies. The common criteria the studies did not meet include: inadequate exposure assessment, loss to follow-up (cohort studies) and low response rates (case-control studies). While we were not able to undertake subgroup analyses by NOS score due to the limited number of studies available, our subgroup analyses by study design showed similar associations between walking and breast cancer in cohort vs. case-control studies.

To our knowledge, this review represents the first systematic attempt to synthesize the existing evidence on specific transport modes and site-specific cancers. We provided mode-specific summary effects where possible and calculated the dose-response effects for transport-related physical activity, in line with WHO physical activity recommendation. When interpreting the findings, some limitations need to be considered. First, the review may not have included some eligible studies published in languages other than English. Second, due to the limited number of available studies, we were not able to pool the results separately for cohort and case-control studies; however, we conducted sub-group analyses by study design where possible. We were not able to evaluate the non-linear relationship between transport-related physical activity and the risks of site-specific cancers. While a recent systematic review on breast and colon cancers reported a linear relationship with physical activity [64], others suggested a non-linear relationship between physical activity and cancer risk [65, 66]. Further, variations in measurement and categorization of the exposure across the studies make direct comparison of the results between different modes (e.g., walking vs. cycling) difficult. Finally, the majority of the studies included were conducted in high income countries in Europe, UK, and North America, limiting the generalizability of the findings to other populations and low and middle income countries where urbanization and motorization are mainly taking place [67].

Our findings suggest that transport choices may influence cancer risk, particularly of obesity-related cancers such as breast, colon and endometrial cancers. Breast cancer is the most common cancer in women globally, with an estimated over 2 million new cases (11.7% of all new cases) in 2020, while colon cancer stood at fourth place (over 1 million cases, 6% of total cases) [68]. The incidence of endometrial cancer also seems to be increasing in many countries particularly in younger women. Our findings indicate that the risks of these cancers can be reduced by meeting the WHO physical activity recommendation through active commuting (∼150 min of walking or 90 min of cycling per week). Yet, the current evidence is limited in relation to other cancer sites, underlying mechanisms, and potential environmental influences, requiring further exploration.

Given heterogeneity in exposure measurements in the existing studies, harmonizing choice of the assessment tool (e.g., using International Physical Activity Questionnaires that can capture information about all four physical activity domains including transport modes), and reporting the dose-response estimates for each transport mode such as walking and cycling separately rather than a combined mode would enhance comparability of results and provide mode-specific effects. Repeated or regular assessments of exposures/transport modes used throughout the study duration would capture changes and their potential impact on outcomes in cohort studies. Importantly, more research is needed in low and middle-income settings to generate context-specific evidence.

In conclusion, active transport modes appear to reduce cancer risk, but evidence for cancer sites other than colorectum, breast and endometrium is currently limited.

### Electronic supplementary material

Below is the link to the electronic supplementary material.

**Supplementary Material 1**: **Supplementary file S1** PRISMA checklist. **Supplementary file S2** Literature search strategy. **Supplementary file S3** List of excluded full texts with reasons. **Supplementary file S4** Metabolic Equivalent of Task (MET) values used and MET hour per week conversion formulas. **Supplementary file S5** Measurement units, effect measures and covariates included in the studies. **Supplementary file S6** Newcastle-Ottawa Score of the studies (cohort studies). **Supplementary file S7** Newcastle-Ottawa Score of the studies (case control studies). **Supplementary file S8** Risks estimates used in the meta-analyses (separate excel sheet). **Supplementary file S9** Forest plots. **Supplementary file S10** Sub-group and covariates adjustment analyses. **Supplementary file S11** Sensitivity analysis. **Supplementary file S12** Funnel plots

## Data Availability

No datasets were generated or analysed during the current study.

## References

[1] Lee IM, Shiroma EJ, Lobelo F, Puska P, Blair SN, Katzmarzyk PT (2012). Effect of physical inactivity on major non-communicable diseases worldwide: an analysis of burden of disease and life expectancy. Lancet.

[2] Ding D, Lawson KD, Kolbe-Alexander TL (2016). The economic burden of physical inactivity: a global analysis of major non-communicable diseases. Lancet.

[3] World Health Organization. Global status report on physical activity 2022.; 2022.

[4] Guthold R, Stevens GA, Riley LM, Bull FC (2018). Worldwide trends in insufficient physical activity from 2001 to 2016: a pooled analysis of 358 population-based surveys with 1·9 million participants. Lancet Global Health.

[5] Berrigan D, Troiano RP, McNeel T, DiSogra C, Ballard-Barbash R (2006). Active transportation increases adherence to activity recommendations. Am J Prev Med.

[6] Dinu M, Pagliai G, Macchi C, Sofi F (2019). Active commuting and multiple Health outcomes: a systematic review and Meta-analysis. Sports Med.

[7] Boniface S, Scantlebury R, Watkins SJ, Mindell JS (2015). Health implications of transport: evidence of effects of transport on social interactions. J Transp Health.

[8] Higgins PAT (2005). Exercise-based transportation reduces oil dependence, carbon emissions and obesity. Envir Conserv.

[9] World Health Organization. Global Status Report on Road Safety 2018: Summary.; 2018.

[10] Sugiyama T, Chandrabose M, Homer AR, Sugiyama M, Dunstan DW, Owen N (2020). Car use and cardiovascular disease risk: systematic review and implications for transport research. J Transp Health.

[11] Panchal R, Panagi M, May HR (2022). Personal air pollution exposure during morning commute car and active transport journeys. J Transp Health.

[12] Turner MC, Andersen ZJ, Baccarelli A (2020). Outdoor air pollution and cancer: an overview of the current evidence and public health recommendations. CA Cancer J Clin.

[13] Moore SC, Lee IM, Weiderpass E (2016). Association of leisure-time physical activity with risk of 26 types of Cancer in 1.44 million adults. JAMA Intern Med.

[14] Rezende LFMD, Sá THD, Markozannes G (2018). Physical activity and cancer: an umbrella review of the literature including 22 major anatomical sites and 770 000 cancer cases. Br J Sports Med.

[15] Hermelink R, Leitzmann MF, Markozannes G (2022). Sedentary behavior and cancer–an umbrella review and meta-analysis. Eur J Epidemiol.

[16] Wells G, Shea B, O’Connell D et al. The Newcastle-Ottawa Scale (NOS) for assessing the quality of nonrandomised studies in meta-analyses. The Newcastle-Ottawa Scale (NOS) for assessing the quality of nonrandomised studies in meta-analyses. https://www.ohri.ca/programs/clinical_epidemiology/oxford.asp.

[17] Taylor K. Wanting a particular reference category in categorical risk data. Data extraction tips for meta-analysis. https://www.cebm.ox.ac.uk/resources/data-extraction-tips-meta-analysis/reference-category-risk-data.

[18] Greenland S, Longnecker MP (1992). Methods for Trend Estimation from Summarized Dose-Response Data, with applications to Meta-Analysis. Am J Epidemiol.

[19] Viechtbauer W. Conducting Meta-analyses in R with the metafor Package. J Stat Soft. 2010;36(3). 10.18637/jss.v036.i03.

[20] Crippa A, Orsini N (2016).

[21] Schouten LJ, Goldbohm RA, van den Brandt PA, Anthropometry (2004). Physical activity, and Endometrial Cancer Risk: results from the Netherlands Cohort Study. JNCI J Natl Cancer Inst.

[22] Biesma RG, Schouten LJ, Dirx MJM, Goldbohm RA, van den Brandt PA (2006). Physical activity and risk of ovarian Cancer: results from the Netherlands Cohort Study (the Netherlands). Cancer Causes Control.

[23] Zeegers MPA, Dirx MJM, van den Brandt PA (2005). Physical activity and the risk of prostate Cancer in the Netherlands Cohort Study, results after 9.3 years of follow-up. Cancer Epidemiol Biomarkers Prev.

[24] Simons CCJM, Hughes LAE, van Engeland M, Goldbohm RA, van den Brandt PA, Weijenberg MP (2013). Physical activity, occupational sitting time, and Colorectal Cancer Risk in the Netherlands Cohort Study. Am J Epidemiol.

[25] Wong JYY, Jones RR, Breeze C (2021). Commute patterns, residential traffic-related air pollution, and lung cancer risk in the prospective UK Biobank cohort study. Environ Int.

[26] Panter J, Mytton O, Sharp S (2018). Using alternatives to the car and risk of all-cause, cardiovascular and cancer mortality. Heart.

[27] Celis-Morales CA, Lyall DM, Welsh P (2017). Association between active commuting and incident cardiovascular disease, cancer, and mortality: prospective cohort study. BMJ Published Online April.

[28] Pronk A, Ji BT, Shu XO (2011). Physical activity and breast cancer risk in Chinese women. Br J Cancer.

[29] Matthews CE, Jurj AL, Shu Xo (2007). Influence of Exercise, walking, Cycling, and overall nonexercise physical activity on Mortality in Chinese women. Am J Epidemiol.

[30] George SM, Irwin ML, Matthews CE (2010). Beyond recreational physical activity: examining Occupational and Household Activity, Transportation Activity, and sedentary behavior in relation to postmenopausal breast Cancer Risk. Am J Public Health.

[31] Gierach GL, Chang SC, Brinton LA (2009). Physical activity, sedentary behavior, and endometrial cancer risk in the NIH-AARP Diet and Health Study. Int J Cancer.

[32] Luoto R, Latikka P, Pukkala E, Hakulinen T, Vihko V (2000). The effect of physical activity on breast cancer risk: a cohort study of 30,548 women. Eur J Epidemiol.

[33] Gomes MLB, Pinto SS, Domingues MR (2022). Physical activity and breast Cancer: a case-control study in Southern Brazil. Nutr Cancer.

[34] Azubuike SO, Hayes L, Sharp L, Alabi A, Oyesegun RA, McNally R (2022). Physical activity and the risk of breast cancer among Nigerian women. Cancer Epidemiol.

[35] Si S, Boyle T, Heyworth J, Glass DC, Saunders C, Fritschi L (2015). Lifetime physical activity and risk of breast cancer in pre-and post-menopausal women. Breast Cancer Res Treat.

[36] Mathew A, Gajalakshmi V, Rajan B (2009). Physical activity levels among urban and rural women in south India and the risk of breast cancer: a case–control study. Eur J Cancer Prev.

[37] Steindorf K (2003). Case-control study of physical activity and breast Cancer risk among Premenopausal women in Germany. Am J Epidemiol.

[38] John EM, Horn-Ross PL, Koo J (2003). Lifetime physical activity and breast cancer risk in a multiethnic population: the San Francisco Bay area breast cancer study. Cancer Epidemiol Biomarkers Prev.

[39] Matthews CE, Shu XO, Jin F (2001). Lifetime physical activity and breast cancer risk in the Shanghai breast Cancer Study. Br J Cancer.

[40] Marcus PM, Newman B, Moorman PG (1999). Physical activity at age 12 and adult breast cancer risk (United States). Cancer Causes Control.

[41] Friberg E, Mantzoros CS, Wolk A (2006). Physical activity and risk of Endometrial Cancer: a Population-based prospective cohort study. Cancer Epidemiol Biomarkers Prev.

[42] John EM, Koo J, Horn-Ross PL (2010). Lifetime physical activity and risk of Endometrial Cancer. Cancer Epidemiol Biomarkers Prev.

[43] Matthews CE, Xu WH, Zheng W (2005). Physical activity and risk of Endometrial Cancer: a report from the Shanghai Endometrial Cancer Study. Cancer Epidemiol Biomarkers Prev.

[44] Mahmood S, English DR, MacInnis RJ (2018). Domain-specific physical activity and the risk of colorectal cancer: results from the Melbourne Collaborative Cohort Study. BMC Cancer.

[45] Hou L (2004). Commuting physical activity and risk of Colon cancer in Shanghai, China. Am J Epidemiol.

[46] Littman AJ, Doody DR, Biggs ML, Weiss NS, Starr JR, Schwartz SM (2009). Physical activity in adolescence and testicular germ cell cancer risk. Cancer Causes Control.

[47] Coldman AJ, Elwood JM, Gallagher RP (1982). Sports activities and risk of testicular cancer. Br J Cancer.

[48] Hosseini M, SeyedAlinaghi S, Mahmoudi M, McFarland W (2010). A case-control study of risk factors for prostate cancer in Iran. Acta Med Iran.

[49] Pang Y, Lv J, Kartsonaki C (2021). Association of physical activity with risk of hepatobiliary diseases in China: a prospective cohort study of 0.5 million people. Br J Sports Med.

[50] Xiao Q, Liao L, Matthews CE (2014). Physical activity and renal cell carcinoma among black and white americans: a case-control study. BMC Cancer.

[51] Patterson R, Panter J, Vamos EP, Cummins S, Millett C, Laverty AA (2020). Associations between commute mode and cardiovascular disease, cancer, and all-cause mortality, and cancer incidence, using linked Census data over 25 years in England and Wales: a cohort study. Lancet Planet Health.

[52] Sahlqvist S, Goodman A, Simmons RK (2013). The association of cycling with all-cause, cardiovascular and cancer mortality: findings from the population-based EPIC-Norfolk cohort. BMJ Open.

[53] Autenrieth CS, Baumert J, Baumeister SE (2011). Association between domains of physical activity and all-cause, cardiovascular and cancer mortality. Eur J Epidemiol.

[54] Batty GD, Shipley MJ, Marmot M, Smith GD (2001). Physical activity and cause-specific mortality in men: further evidence from the Whitehall study. Eur J Epidemiol.

[55] Wu Y, Zhang D, Kang S (2013). Physical activity and risk of breast cancer: a meta-analysis of prospective studies. Breast Cancer Res Treat.

[56] Moore SC, Gierach GL, Schatzkin A, Matthews CE (2010). Physical activity, sedentary behaviours, and the prevention of endometrial cancer. Br J Cancer.

[57] García-Estévez L, Cortés J, Pérez S, Calvo I, Gallegos I, Moreno-Bueno G (2021). Obesity and breast Cancer: a paradoxical and controversial relationship influenced by Menopausal Status. Front Oncol.

[58] Chan DSM, Abar L, Cariolou M (2019). World Cancer Research Fund International: continuous update project—systematic literature review and meta-analysis of observational cohort studies on physical activity, sedentary behavior, adiposity, and weight change and breast cancer risk. Cancer Causes Control.

[59] Compedium of Physical Activities. 17-Walking. Accessed August 3. 2023. https://sites.google.com/site/compendiumofphysicalactivities/Activity-Categories/walking?authuser=0.

[60] Mahmood S, MacInnis RJ, English DR, Karahalios A, Lynch BM (2017). Domain-specific physical activity and sedentary behaviour in relation to colon and rectal cancer risk: a systematic review and meta-analysis. Int J Epidemiol.

[61] Samad AKA, Taylor RS, Marshall T, Chapman MAS (2005). A meta-analysis of the association of physical activity with reduced risk of colorectal cancer. Colorect Dis.

[62] McTiernan A (2008). Mechanisms linking physical activity with cancer. Nat Rev Cancer.

[63] Peters HPF (2001). Potential benefits and hazards of physical activity and exercise on the gastrointestinal tract. Gut.

[64] Diao X, Ling Y, Zeng Y (2023). Physical activity and cancer risk: a dose-response analysis for the global burden of Disease Study 2019. Cancer Commun.

[65] Garcia L, Pearce M, Abbas A (2023). Non-occupational physical activity and risk of cardiovascular disease, cancer and mortality outcomes: a dose–response meta-analysis of large prospective studies. Br J Sports Med.

[66] Li T, Wei S, Shi Y (2016). The dose–response effect of physical activity on cancer mortality: findings from 71 prospective cohort studies. Br J Sports Med.

[67] Unite, Nations. Department of Economic and Social Affair. World urbanization prospects: the 2018 revision. United Nations; 2019.

[68] Sung H, Ferlay J, Siegel RL, Cancer (2021). J Clin.

