# Supplementary material for: Associations between transport modes and site-specific cancers: a systematic review and meta-analysis
Source: Environ Health. 2024 Apr 13;23:39. doi: 10.1186/s12940-024-01081-3 (PMC11015678; doi:10.1186/s12940-024-01081-3)
Supplement: Supplementary file 1 — Supplementary Material 1: Supplementary file S1 PRISMA checklist. Supplementary file S2 Literature search strategy. Supplementary file S3 List of excluded full texts with reasons. Supplementary file S4 Metabolic Equivalent of Task (MET) values used and MET hour per week conversion formulas. Supplementary file S5 Measurement units, effect measures and covariates included in the studies. Supplementary file S6 Newcastle-Ottawa Score of the studies (cohort studies). Supplementary file S7 Newcastle-Ottawa Score of the studies (case control studies). Supplementary file S8 Risks estimates used in the meta-analyses (separate excel sheet). Supplementary file S9 Forest plots. Supplementary file S10 Sub-group and covariates adjustment analyses. Supplementary file S11 Sensitivity analysis. Supplementary file S12 Funnel plots [file 12940_2024_1081_MOESM1_ESM.docx]

**Supplementary files**

**Table of Content**

S1: PRISMA checklist……………………………………………………………………………2

S2: Literature search strategy…………………………………………………………………….5

S3: List of excluded full texts with reasons……………………………………………………...7

S4: Metabolic Equivalent of Task (MET) values used and MET hour per week conversion formulas………………………………………………………………………………………….9

S5: Measurement units, effect measures and covariates in the studies………………………….10

S6: Newcastle-Ottawa Score of the studies (cohort studies)…………………………………….21

S7: Newcastle-Ottawa Score of the studies (case control studies)………………………………22

S8: Risks estimates used in the meta-analyses (separate excel sheet)…………………………...23

S9: Forest plots…………………………………………………………………………………24

S10: Sub-group and covariates adjustment analyses……………………………………….......30

S11: Sensitivity analysis………………………………………………………………………..31

S12: Funnel plots………………………………………………………………………………33

**Supplementary file 1: PRISMA checklist**

| **Section and Topic** | **Item #** | **Checklist item** | **Location where item**  **is reported** |  |
| --- | --- | --- | --- | --- |
| **TITLE** | | |  |  |
| Title | 1 | Identify the report as a systematic review. | Title |  |
| **ABSTRACT** | | |  |  |
| Abstract | 2 | See the PRISMA 2020 for Abstracts checklist. | Abstract |  |
| **INTRODUCTION** | | |  |  |
| Rationale | 3 | Describe the rationale for the review in the context of existing knowledge. | Introduction |  |
| Objectives | 4 | Provide an explicit statement of the objective(s) or question(s) the review addresses. | Introduction, last para |  |
| **METHODS** | | |  |  |
| Eligibility criteria | 5 | Specify the inclusion and exclusion criteria for the review and how studies were grouped for the syntheses. | Search strategy and study selection |  |
| Information sources | 6 | Specify all databases, registers, websites, organisations, reference lists and other sources searched or consulted to identify studies. Specify the date when each source was last searched or consulted. | Search strategy and study selection |  |
| Search strategy | 7 | Present the full search strategies for all databases, registers and websites, including any filters and limits used. | Supplementary file S2 |  |
| Selection process | 8 | Specify the methods used to decide whether a study met the inclusion criteria of the review, including how many reviewers screened each record and each report retrieved, whether they worked independently, and if applicable, details of automation tools used in the process. | Search strategy and study selection section |  |
| Data collection process | 9 | Specify the methods used to collect data from reports, including how many reviewers collected data from each report, whether they worked independently, any processes for obtaining or confirming data from study investigators, and if applicable, details of automation tools used in the process. | Data extraction and study quality assessment section |  |
| Data items | 10a | List and define all outcomes for which data were sought. Specify whether all results that were compatible with each outcome domain in each study were sought (e.g. for all measures, time points, analyses), and if not, the methods used to decide which results to collect. | Data extraction and study quality assessment section |  |
|  | 10b | List and define all other variables for which data were sought (e.g. participant and intervention characteristics, funding sources). Describe any assumptions made about any missing or unclear information. | Data extraction and study quality assessment section |  |
| Study risk of bias assessment | 11 | Specify the methods used to assess risk of bias in the included studies, including details of the tool(s) used, how many reviewers assessed each study and whether they worked independently, and if applicable, details of automation tools used in the process. | Data extraction and study quality assessment section |  |
| Effect measures | 12 | Specify for each outcome the effect measure(s) (e.g. risk ratio, mean difference) used in the synthesis or presentation of results. | Data extraction and quality assessment section para |  |
| Synthesis methods | 13a | Describe the processes used to decide which studies were eligible for each synthesis (e.g. tabulating the study intervention characteristics and comparing against the planned groups for each synthesis (item #5)). | Data analysis  para 1 |  |
|  | 13b | Describe any methods required to prepare the data for presentation or synthesis, such as handling of missing summary statistics, or data conversions. | Data analysis  para 1 |  |
|  | 13c | Describe any methods used to tabulate or visually display results of individual studies and syntheses. | NA |  |
|  | 13d | Describe any methods used to synthesize results and provide a rationale for the choice(s). If meta-analysis was performed, describe the model(s), method(s) to identify the presence and extent of statistical heterogeneity, and software package(s) used. | Data analysis  para 1 |  |
|  | 13e | Describe any methods used to explore possible causes of heterogeneity among study results (e.g. subgroup analysis, meta-regression). | Data analysis  Para 2 |  |
|  | 13f | Describe any sensitivity analyses conducted to assess robustness of the synthesized results. | Data analysis  para 2 |  |
| Reporting bias assessment | 14 | Describe any methods used to assess risk of bias due to missing results in a synthesis (arising from reporting biases). | NA |  |
| Certainty assessment | 15 | Describe any methods used to assess certainty (or confidence) in the body of evidence for an outcome. | NA |  |
| **Section and Topic** | **Item #** | **Checklist item** | **Location where item**  **is reported** | |
| **RESULTS** | | |  | |
| Study selection | 16a | Describe the results of the search and selection process, from the number of records identified in the search to the number of studies included in the review, ideally using a flow diagram. | Fig 1  Result section, para 1 | |
|  | 16b | Cite studies that might appear to meet the inclusion criteria, but which were excluded, and explain why they were excluded. | Supplementary file S3 | |
| Study characteristics | 17 | Cite each included study and present its characteristics. | Table 1 | |
| Risk of bias in studies | 18 | Present assessments of risk of bias for each included study. | Presented qualities assessed for each study in Supple file S6 | |
| Results of individual studies | 19 | For all outcomes, present, for each study: (a) summary statistics for each group (where appropriate) and (b) an effect estimate and its precision (e.g. confidence/credible interval), ideally using structured tables or plots. | S5, S7 | |
| Results of syntheses | 20a | For each synthesis, briefly summarise the characteristics and risk of bias among contributing studies. | Result section  Para 4-11 | |
|  | 20b | Present results of all statistical syntheses conducted. If meta-analysis was done, present for each the summary estimate and its precision (e.g. confidence/credible interval) and measures of statistical heterogeneity. If comparing groups, describe the direction of the effect. | Fig 3 | |
|  | 20c | Present results of all investigations of possible causes of heterogeneity among study results. | Result section para 12,  S8 | |
|  | 20d | Present results of all sensitivity analyses conducted to assess the robustness of the synthesized results. | Result section para 12, S9 | |
| Reporting biases | 21 | Present assessments of risk of bias due to missing results (arising from reporting biases) for each synthesis assessed. | NA | |
| Certainty of evidence | 22 | Present assessments of certainty (or confidence) in the body of evidence for each outcome assessed. | NA | |
| **DISCUSSION** | | |  | |
| Discussion | 23a | Provide a general interpretation of the results in the context of other evidence. | Discussion section  para 2,3 | |
|  | 23b | Discuss any limitations of the evidence included in the review. | Discussion section  para 5 | |
|  | 23c | Discuss any limitations of the review processes used. | Discussion section  para 5 | |
|  | 23d | Discuss implications of the results for practice, policy, and future research. | Discussion section  para 6,7 | |
| **OTHER INFORMATION** | | |  | |
| Registration and protocol | 24a | Provide registration information for the review, including register name and registration number, or state that the review was not registered. | Methods section, para 1 | |
|  | 24b | Indicate where the review protocol can be accessed, or state that a protocol was not prepared. | NA | |
|  | 24c | Describe and explain any amendments to information provided at registration or in the protocol. | NA | |
| Support | 25 | Describe sources of financial or non-financial support for the review, and the role of the funders or sponsors in the review. | Role of funding section | |
| Competing interests | 26 | Declare any competing interests of review authors. | Declaration of interest | |
| Availability of data, code and other materials | 27 | Report which of the following are publicly available and where they can be found: template data collection forms; data extracted from included studies; data used for all analyses; analytic code; any other materials used in the review. | Supplementary file S6 | |

**Supplementary file S2. Literature search strategy**

| **PubMed (1914 to 17^th^ February 2023)**  ("public transport"[Title] OR "bus"[Title] OR "train"[Title]) AND "health"[Title]  ("public transport"[Title] OR "bus"[Title] OR "train"[Title]) AND "cancer"[Title]  ("active travel"[Title] OR "active transport"[Title]) AND "cancer"[Title]  ("car"[Title] OR "motor vehicle"[Title] OR "driving"[Title]) AND "cancer"[Title]  "commut*"[Title] AND "cancer"[Title]  (("walk"[Title] OR "cycle"[Title] OR "public transport"[Title] OR "bus"[Title] OR train [Title] OR "car"[Title] OR "physical activity"[Title]) AND "cancer risk"[Title])  ("physical activity"[Title] OR "walking"[Title] OR "cycling"[Title] OR "bicycle"[Title]) AND "bladder cancer"[Title]  "physical activity"[Title] AND "breast cancer"[Title]  ("walking"[Title] OR "cycling"[Title] OR "bicycle"[Title] OR "physical activity"[Title]) AND "colon cancer"[Title]  ("walking"[Title] OR "cycling"[Title] OR "bicycle"[Title] OR "physical activity"[Title]) AND "colorectal cancer"[Title]  ("walking"[Title] OR "cycling"[Title] OR "bicycle"[Title] OR "physical activity"[Title]) AND "gastrointestinal cancer"[Title]  ("walking"[Title] OR "cycling"[Title] OR "bicycle"[Title] OR "physical activity"[Title]) AND "rectal cancer"[Title]  ("walking"[Title] OR "cycling"[Title] OR "bicycle"[Title] OR "physical activity"[Title]) AND "endometrial cancer"[Title]  "physical activity"[Title] AND "oesophageal cancer"[Title]  ("physical activity"[Title] AND ("oesophagus"[All Fields] OR "esophagus"[MeSH Terms] OR "esophagus"[All Fields])) OR "esophagus adenocarcinoma"[Title]  (("walking"[Title] OR "bicycle"[Title] OR "cycling"[Title] OR "physical activity"[Title]) AND "oesophagus"[Title]) OR "esophagus cancer"[Title]  ("walking"[Title] OR "cycling"[Title] OR "bicycle"[Title] OR "physical activity"[Title]) AND "gastric adenocarcinoma"[Title]  ("walking"[Title] OR "cycling"[Title] OR "bicycle"[Title] OR "physical activity"[Title]) AND "stomach cancer"[Title]  ("walking"[Title] OR "cycling"[Title] OR "bicycle"[Title] OR "physical activity"[Title]) AND "gastric cancer"[Title]  ("walking"[Title] OR "cycling"[Title] OR "bicycle"[Title] OR "physical activity"[Title]) AND "hepatobilliary cancer"[Title]  ("walking"[Title] OR "cycling"[Title] OR "bicycle"[Title] OR "physical activity"[Title]) AND "liver cancer"[Title]  "physical activity"[Title] AND "lung cancer"[Title]  ("walking"[Title] OR "cycling"[Title] OR "bicycle"[Title] OR "physical activity"[Title]) AND "lymphoma"[Title]  ("walking"[Title] OR "cycling"[Title] OR "bicycle"[Title] OR "physical activity"[Title]) AND "melanoma"[Title]  ("walking"[Title] OR "cycling"[Title] OR "bicycle"[Title] OR "physical activity"[Title]) AND "glioma"[Title]  ("walking"[Title] OR "cycling"[Title] OR "bicycle"[Title] OR "physical activity"[Title]) AND "meningioma"[Title]  ("walking"[Title] OR "cycling"[Title] OR "bicycle"[Title] OR "physical activity"[Title]) AND "myeloma"[Title]  ("walking"[Title] OR "cycling"[Title] OR "bicycle"[Title] OR "physical activity"[Title]) AND "ovarian cancer"[Title]  ("walking"[Title] OR "cycling"[Title] OR "bicycle"[Title] OR "physical activity"[Title]) AND "pancreatic cancer"[Title]  ("walking"[Title] OR "cycling"[Title] OR "bicycle"[Title] OR "physical activity"[Title]) AND "prostate cancer"[Title]  ("walking"[Title] OR "cycling"[Title] OR "bicycle"[Title] OR "physical activity"[Title]) AND "renal cell cancer"[Title]  ("walking"[Title] OR "cycling"[Title] OR "bicycle"[Title] OR "physical activity"[Title]) AND "renal cell carcionma"[Title]  ("walking"[Title] OR "cycling"[Title] OR "bicycle"[Title] OR "physical activity"[Title]) AND "kidney cancer"[Title]  ("walking"[Title] OR "cycling"[Title] OR "bicycle"[Title] OR "physical activity"[Title]) AND "testicular cancer"[Title]  ("walking"[Title] OR "cycling"[Title] OR "bicycle"[Title] OR "physical activity"[Title]) AND "thyroid cancer"[Title]  ("walking"[Title] OR "cycling"[Title] OR "bicycle"[Title] OR "physical activity"[Title]) AND "breast carcinoma"[Title]  ("walking"[Title] OR "cycling"[Title] OR "bicycle"[Title] OR "physical activity"[Title]) AND "endometrial adeno carcinoma"[Title] |
| --- |
| **Embase (1980 – 17^th^ February 2023)**  ((Public transport or bus or train) and health).m_titl.  ((Public transport or bus or train) and cancer).m_titl.  ((active travel or active transport) and cancer).m_titl.  ((car or motor vehicle or driving) and cancer).m_titl.  (commute and cancer).m_titl.  ((walk or cycle or public transport or bus or train or car or physical activity) and cancer risk).m_titl.  ((walking or cycling or bicycle or physical activity) and bladder cancer).m_titl.  (physical activity and breast cancer).m_titl.  ((walking or cycling or bicycle or physical activity) and colon cancer).m_titl.  ((walking or cycling or bicycle or physical activity) and colorectal cancer).m_titl.  ((walking or cycling or bicycle or physical activity) and gastrointestinal cancer).m_titl.  ((walking or cycling or bicycle or physical activity) and rectal cancer).m_titl.  ((walking or cycling or bicycle or physical activity) and endometrial cancer).m_titl.  (physical activity and oesophageal cancer).m_titl.  (physical activity and oesophagus or esophagus cancer).m_titl.  (((walking or bicycle or cycling or physical activity) and oesophagus) or esophagus cancer).m_titl.  ((walking or cycling or bicycle or physical activity) and gastric adenocarcinoma).m_titl.  ((walking or cycling or bicycle or physical activity) and gastric cancer).m_titl.  ((walking or cycling or bicycle or physical activity) and stomach caner).m_titl.  ((walking or cycling or bicycle or physical activity) and liver cancer).m_titl.  ((walking or cycling or bicycle or physical) and activity and hepatobiliary diseases).m_titl.  (physical activity and lung cancer).m_titl.  ((walking or cycling or bicycle or physical activity) and lymphoma).m_titl  ((walking or cycling or bicycle or physical activity) and melanoma).m_titl  ((walking or cycling or bicycle or physical activity) and glioma).m_titl  ((walking or cycling or bicycle or physical activity) and meningioma).m_titl  ((walking or cycling or bicycle or physical activity) and myeloma).m_titl  ((walking or cycling or bicycle or physical activity) and ovarian cancer).m_titl  ((walking or cycling or bicycle or physical activity) and pancreatic cancer).m_titl  ((walking or cycling or bicycle or physical activity) and prostate cancer).m_titl  ((walk or cycling or bicycle or physical activity) and renal cell cancer).m_titl  ((walk or cycling or bicycle or physical activity) and renal cell carcinoma).m_titl  (walking or cycling or bicycle or physical activity) and kidney cancer).m_titl  ((walking or cycling or bicycle or physical activity) and testicular cancer).m_titl.  ((walking or cycling or bicycle or physical activity) and testicular cancer).m_titl.  ((walking or cycling or bicycle or physical activity) and breast carcinoma).m_titl.  ((walking or cycling or bicycle or physical activity) and endometrial adenocarcinoma).m_titl. |
| **Scopus (1914 to 17^th^ February 2023)**  TITLE ( public AND transport OR bus OR train AND health )  TITLE ( public AND transport OR bus OR train AND cancer)  TITLE ( active AND travel OR active AND transport AND cancer )  TITLE ( car OR motor vehicle OR driving AND cancer )  TITLE ( commute AND cancer )  TITLE ( walk OR cycle OR public AND transport OR bus OR train OR car OR physical AND activity AND cancer AND risk )  TITLE ( walk OR cycle OR bicycle OR physical AND activity AND bladder AND cancer )  TITLE (physical AND activity AND breast AND cancer )  TITLE ( walking OR cycling OR bicycle OR physical AND activity AND colon AND cancer )  TITLE ( walking OR cycling OR bicycle OR physical AND activity AND colorectal AND cancer )  TITLE ( walking OR cycling OR bicycle OR physical AND activity AND gastrointestinal AND cancer )  TITLE ( walking OR cycling OR bicycle OR physical AND activity AND rectal AND cancer )  TITLE ( walking OR cycling OR bicycle OR physical AND activity AND endometrial AND cancer )  TITLE (physical AND activity AND esophageal AND cancer )  TITLE ( physical AND activity AND oesophagus OR esophagus AND adenocarcinoma )  TITLE ( walk OR bicycle OR cycling OR physical AND activity AND oesophagus OR esophagus AND cancer )  TITLE ( walk OR bicycle OR cycling OR physical AND activity AND gastric AND adenocarcinoma)  TITLE ( walking OR cycling OR bicycle OR physical AND activity AND gastric AND cancer )  TITLE ( walking OR cycling OR bicycle OR physical AND activity AND stomach AND cancer )  TITLE ( walking OR cycling OR bicycle OR physical AND activity AND hepatobiliary AND cancer )  TITLE ( walking OR cycling OR bicycle OR physical AND activity AND liver AND cancer )  TITLE (physical AND activity AND lung AND cancer )  TITLE ( walking OR cycling OR bicycle OR physical AND activity AND lymphoma)  TITLE ( walking OR cycling OR bicycle OR physical AND activity AND melanoma)  TITLE ( walking OR cycling OR bicycle OR physical AND activity AND glioma)  TITLE ( walking OR cycling OR bicycle OR physical AND activity AND meningioma)  TITLE ( walking OR cycling OR bicycle OR physical AND activity AND myeloma)  TITLE ( walking OR cycling OR bicycle OR physical AND activity AND ovarian AND cancer)  TITLE ( walking OR cycling OR bicycle OR physical AND activity AND pancreatic AND cancer)  TITLE ( walking OR cycling OR bicycle OR physical AND activity AND prostate AND cancer)  TITLE ( walking OR cycling OR bicycle OR physical AND activity AND renal AND cell AND cancer)  TITLE ( walking OR cycling OR bicycle OR physical AND activity AND renal AND cell AND carcinoma)  TITLE ( walking OR cycling OR bicycle OR physical AND activity AND kidney AND cancer)  TITLE ( walking OR cycling OR bicycle OR physical AND activity AND testicular AND cancer)  TITLE ( walking OR cycling OR bicycle OR physical AND activity AND thyroid AND cancer)  TITLE ( walking OR cycling OR bicycle OR physical AND activity AND breast AND carcinoma )  TITLE ( walking OR cycling OR bicycle OR physical AND activity AND endometrial AND adenocarcinoma ) |

**Supplementary file 3: Excluded publications with reasons**

| **Author, year** | **Title** | **Reason** |
| --- | --- | --- |
| Su et al., 2022 | Association between physical activity and cancer risk among Chinese adults: a 10-year prospective study | Commuting PA is included but risk associated is not provided separately |
| Perrier et al., 2022 | Life-Course Trajectories of Physical Activity and Melanoma Risk in a Large Cohort of Norwegian Women | Commuting PA is included but risk associated is not provided separately |
| Huerta et al., 2019 | Domain-specific patterns of physical activity and risk of breast cancer sub-types in the MCC-Spain study | Transport domain specific risk is not provided |
| Steindorf et al., 2013 | Physical activity and risk of breast cancer overall and by hormone receptor status: The European prospective investigation into cancer and nutrition | Walking and cycling for transport are included in PA but not provided associated specific risks |
| Heinen et al., 2011 | Physical activity, energy restriction, and the risk of pancreatic cancer: Prospective study in the Netherlands | Walking and cycling for transport are included in PA but not provided associated specific risks |
| Steindorf et al., 2005 | Case-control study of lifetime occupational and recreational physical activity and risks of colon and rectal cancer | Walking and cycling for transport are included in PA, not provide risks |
| Van Dijk et al., 2004 | Relation of height, body mass, energy intake, and physical activity to risk of renal cell carcinoma: Results from the Netherlands Cohort Study | Walking and cycling for transport are included in PA but not provided associated specific risks |
| Friedenreich et al., 2001 | Influence of physical activity in different age and life periods on the risk of breast cancer | Walking or cycling for transport is included in PA, but not provided associated risk |
| Colditz et al., 2003 | Physical activity and risk of breast cancer in premenopausal women | Walking included is not specific for transport |
| Suzuki et al., 2008 | Effect of Physical Activity on Breast Cancer Risk: Findings of the Japan Collaborative Cohort Study | Domain associated with walking was not specified |
| Zhang et al., 2015 | Adult body size and physical activity in relation to risk of breast cancer according to tumor androgen receptor status | Domain associated with walking was not specified |
| Chen et al.,2022 | Physical activity, polygenic risk score, and colorectal cancer risk | Domains associated with walking and cycling were not specified |
| Howard et al.,2008 | Physical activity, sedentary behavior, and the risk of colon and rectal cancer in the NIH-AARP Diet and Health Study | Domain associated with walking was not specified |
| Lee et al.,2007 | Physical activity and risk of colorectal cancer in Japanese men and women: the Japan Public Health Center-based prospective Study | Domain associated with walking was not specified |
| Takahashi et al.,2007 | Time spent walking and risk of colorectal cancer in Japan: The Miyagi Cohort Study | Domain associated with walking was not specified |
| Du et al.,2014 | Physical Activity and Risk of Endometrial Adenocarcinoma in the Nurses’ Health Study | Domain associated with walking was not specified |
| Levi et al.,1993 | Selected physical activities and the risk of endometrial cancer | Domain associated with walking was not specified |
| Miyata et al.,2021 | Associations of Body Mass Index, Weight Change, Physical Activity, and Sedentary Behavior With Endometrial Cancer Risk Among Japanese Women: The Japan Collaborative Cohort Study | Domain associated with walking was not specified |
| Huerta et al.,2010 | Prospective study of physical activity and risk of primary adenocarcinomas of the oesophagus and stomach in the EPIC (European Prospective Investigation into Cancer and nutrition) cohort | Walking and cycling were not transport specific |
| Kunzmann et al.,2018 | Physical activity, sedentary behaviour and risk of oesophago-gastric cancer: A prospective cohort study within UK Biobank | Domain associated with walking was not specified |
| Huerta et al.,2017 | Physical activity domains and risk of gastric adenocarcinoma in the MCC-Spain case-control study | Walking and cycling included were not specific for transport |
| Ukawa et al.,2014 | Associations of daily walking and television viewing time with liver cancer mortality: findings from the Japan Collaborative Cohort Study | Domain associated with walking was not specified |
| Orsini et al.,2008 | Association of physical activity with cancer incidence, mortality, and survival: a population-based study of men | Domains associated with walking and cycling were not specified |
| Inoue et al., 2008 | Daily Total Physical Activity Level and Total Cancer Risk in Men and Women: Results from a Large-scale Population-based Cohort Study in Japan | Domain associated with walking was not specified |
| Lee et al., 1999 | Physical activity and risk of lung cancer | Domain associated with walking was not specified |
| Sprague et al., 2008 | Physical activity, white blood cell count, and lung cancer risk in a prospective cohort study | Domain associated with walking was not specified |
| Khan et al.,2006 | Risk Factors for Multiple Myeloma: Evidence from the Japan Collaborative Cohort (JACC) Study | Domain associated with walking was not specified |
| Kaskel et al.,2001 | Outdoor activities in childhood: a protective factor for cutaneous melanoma? Results of a case control study in 271 matched pairs | Domains associated with walking and cycling were not specified |
| Perrier et al.,2021 | Physical activity and cutaneous melanoma risk: A Norwegian population-based cohort study | Domain associated with walking was not specified |
| Gregg et al.,2003 | Relationship of Changes in Physical Activity and Mortality Among Older Women | Domain associated with walking was not specified |
| Hakim et al.,1998 | Effects of walking on mortality among nonsmoking retired men | Domain associated with walking was not specified |
| Patel et al.,2018 | Walking in Relation to Mortality in a Large Prospective Cohort of Older U.S. Adults | Domain associated with walking was not specified |
| Chionh et al.,2010 | Physical activity, body size and composition, and risk of ovarian cancer | Walking was not transport specific (non-occupational) |
| Xiao et al.,2013 | Physical activity in different periods of life, sedentary behavior and the risk of ovarian cancer in the NIH-AARP Diet and Health Study | Domain associated with walking was not specified |
| Wang et al., 2021 | Early life physical activity and risk of ovarian cancer in adulthood | walking for transport is included, but risk associated is not provided separately |
| Lee et al., 2003 | Physical activity, body weight, and pancreatic cancer mortality | Domain associated with walking was not specified |
| Michaud et al., 2001 | Physical Activity, Obesity, Height, and the Risk of Pancreatic Cancer | Domain associated with walking was not specified |
| Lin et al., 2007 | Obesity, physical activity and the risk of pancreatic cancer in a large Japanese cohort | Domain associated with walking was not specified |
| Reulen et al.,2017 | Physical activity and risk of prostate and bladder cancer in China: The South and East China case-control study on prostate and bladder cancer | Domains associated with walking and cycling were not specified |
| Orsini et al.,2009 | A prospective study of lifetime physical activity and prostate cancer incidence and mortality | Domains associated with walking or cycling were not specified |
| Wannamethee et al.,2001 | Physical activity and risk of cancer in middle-aged men | Domain associated with walking was not specified |
| Moore et al.,2008 | Physical Activity during Adulthood and Adolescence in Relation to Renal Cell Cancer | Domain associated with walking was not specified |
| Li et al.,2020 | Physical activity and risk of bladder cancer among postmenopausal women | Domain associated with walking was not specified |
| Fiore et al., 2019 | Physical Activity and Thyroid Cancer Risk: A Case-Control Study in Catania (South Italy) | Domain associated with walking was not specified |

PA = physical activity

**Supplementary file 4: Metabolic Equivalent of Task (MET) values**^[[1]](#footnote-1)^ **and MET hour per week conversion**

| **Mode** | **MET value** |
| --- | --- |
| Walking | 4 |
| Cycling | 6.8 |
| Walking and/or cycling = (walking + cycling)/2 | 5.4 |
| **MET hour per week conversion**  x minute per day = (x * MET (mode) * 7)/60  x hour per week = x * MET (mode) |  |

Note: The MET values provided for walking, cycling, and combined walking and cycling are for travel to work.

**Supplementary file 5: Characteristics of included studies**

| **Authors, year, study name if available** | **Age (mean/range), outcome verification** | **Mode*, measurement unit, PA measurement tool and validation (if mentioned), PA period asked** | **Effect measures/relative risk (95% CI)** | **Covariates** |
| --- | --- | --- | --- | --- |
| **Breast cancer** |  |  |  |  |
| Panter et al. 2018  UK Biobank (UKB) | 40-69 years,  Cancer registries | Active patterns of commuting** (compared to car mode) | 1.04 (0.91, 1.19) | Age, sex, ethnicity, urban/rural, area-level deprivation, education, occupation, household income, cars owned, sleep time, screen time. fresh fruit, raw vegetables, cooked vegetables, smoking, PA at work, strenuous sport duration, other exercise duration, leisure walking duration, DIY duration, shift work, alcohol consumption, longstanding limiting illness/disability, BMI |
| Pronk et al.  2011  Shanghai Women Health Study | 40-70 years, Medical records | Walking and cycling separately  MET hour per week (MET h/wk), validation of the tool (yes), previous year | Walking: 0·95 (0·77,1·16)  Cycling: 0·89 (0·63,1·25) | Age, education, family history of breast cancer, age at first birth, and number of pregnancies |
| George et al.  2010,  NIH-AARP (National Institutes of Health-formerly the American Association of Retired Persons) Diet and Health Study | 50-71 years,  Medical records | Walking and cycling combined mode  Duration (years commuted), previous year | Postmenopausal: 0·86 (0·67,1·11) | Age, energy intake, recreational moderate–vigorous physical activity, parity or age at first live birth, menopausal hormone therapy use, number of breast biopsies, smoking, alcohol intake in grams per day, race, education, Body Mass Index (BMI) |
| Luoto et al. 2000 | 15-64 years | Walking and cycling combined mode  Duration (minute per day – min/day) | 0·87 (0·62,1·24) | Physical activity at leisure time,  education, parity, BMI |
| Gomes et al.  2022 | mean age: 60 year  Hospital record | Commuting PA, lifetime total physical activity questionnaire, validation of the tool (yes), lifetime | 1·34 (0·77,2·31) | Family history, age of menarche, hormonal therapy, age of the first child and breastfeeding, smoking, monthly consumption of pork and fish. |
| Azubuike et al.,  2022, Nigeria | 20-82 years, Hospital records | Walking only  MET hr/week, previous year | 0.84 (0.47-1.35) | Age, study sites, ethnicity, family history of breast cancer, additionally adjusted for income, urbanicity, education, menopausal status, total months of breastfeeding, BMI, Household PA and occupational PA |
| Si et al.  2015, Australia, Breast Cancer Employment and Environment Study | 18-80 years, Cancer registries | Transport related physical activity,  MET h/wk, modified Chasan-Taber Physical Activity Questionnaire, lifetime | Premenopausal: 1.34 (0.89,2.00)  Postmenopausal:  1.03 (0.8,1.33) | Age, menopausal status, family history of breast cancer, education levels, type of HRT, age at menarche, age at first birth, and other types of physical activity, breast feeding |
| Mathew et al.  2009 | Hospital records  (provided age groups: <= 34 to >64 years) | Walking only  duration (min/day), previous year | Premenopausal: 0.77 (0.56,1.05)  Postmenopausal: 0.66 (0.46,0.95) | Age and center, religion, marital status, education, socioeconomic status, residence status, body mass index, waist and hip sizes, parity, age at first child- birth, and duration of breast feeding and other PA variables |
| Steindorf et al. 2003 | mean age: cases – 41.9 year,  Controls – 42.5 year  Hospital records | Walking and cycling separately  MET h/wk | Walking: 0.93 (0.63,1.39)  Cycling: 0.66 (0.45,0.97) | First degree family history of breast cancer, number of full-term pregnancies, height, change in BMI, total months of breastfeeding and mean daily alcohol consumption |
| John et al. 2003 | 35-79 years, Cancer registry | Walking and cycling combined mode  Duration (hours/week) | Premenopausal: 0·73 (0·51,1·03)  Postmenopausal: 1·03 (0·81,1·3) | Age, race/ethnicity, country of birth, education, family history of breast cancer, prior biopsy for benign breast disease, age at menarche, parity, age at first full-term pregnancy, breast- feeding, BMI, and other components of total activity. |
| Matthews et al.  2001, China  Shanghai Breast Cancer Study | 25-64 years, Cancer registry | Walking and cycling separately  Duration (min/day)  adolescencee and 10 years before the assessment (adulthood) | Walking (adult):  1·00 (0·78,1·28)  Cycling: 0·97 (0·77,1·22) | Age, education, household income, first-degree family history of breast cancer, history of breast fibroadenoma, age at menarche, age at first live birth, and age at menopause. |
| Marcus et al.  1999, | 20-70 years  Cancer registry | Walking and cycling separately  distance (mile), validation of the tool (yes) | Walking: 0.8 (0.6,1.2)  Cycling: 1.00 (0.7,1.5) | Race and age at diagnosis/selection, sampling design. |
| **Endometrial** |  |  |  |  |
| Gierach et al. 2009  NIH-AARP Diet and Health Study | 50-71 years, Cancer registery | Walking and cycling combined mode Duration (number of years) | 0.66 (0.38, 1.15) | Age, race, smoking status, parity, ever use of oral contraceptives, age at menopause, and hormone therapy formulation, BMI |
| Friberg et al. 2006 | 39-70 years  Cancer registry | Walking and cycling combined mode  duration (hr/day) | 0.71 (0.45, 1.1) | Age in months, parity, history of diabetes, total fruit and vegetable, education, and work/occupation, walking/bicycling, household work, leisure time activity, and leisure time inactivity (watching TV/sitting) simultaneously, BMI |
| Schouten et al. 2004 | 55-69 years  Cancer and pathology registries | Walking and cycling combined mode  Duration (min/day) | 0.5 (0.29, 0.86) | Age, body mass index, age at menarche, use of oral contraceptives, age at menopause, parity, and cigarette smoking. |
| John et al. 2010 | 35-79 years  Cancer registry | Walking /cycling combined mode  Duration (hr/week), lifetime | 0.8 (0.57, 1.13) | Age, race/ethnicity, education, family history of endometrial cancer, age at menarche, full-term pregnancies, duration of oral contraceptive use, duration of hormonal therapy use, menopausal status, BMI, and height. |
| Matthews et al. 2005 | 30-69 years  Cancer registry | Walking and cycling separately  Duration (min/day) | Walk: 0.64 (0.47, 0.87)  Cycling: 1.24 (0.86, 1.81) | Age, age at menarche, menopausal status and age, number of pregnancies, oral contraceptive use, current smoking, ever drinking, family history of cancer, education, height, and body mass index. |
| **Colorectal** |  |  |  |  |
| Panter et al. 2018  UKB | 40-69 years, Cancer registries | Active patterns of commuting** (compared to car mode) | Colon: 1.18 (0.93, 1.5) | Age, sex, ethnicity, urban/rural, area-level deprivation, education, occupation, household income, cars owned, sleep time, screen time. fresh fruit, raw vegetables, cooked vegetables, smoking, PA at work, strenuous sport duration, other exercise duration, leisure walking duration, DIY duration, shift work, alcohol consumption, longstanding limiting illness/disability, BMI |
| Mahmood et al. 2018  Melbourne Collaborative Cohort Study | 40-69 years, Cancer registry | Transport related PA, MET h/wk,  long-form of International Physical Activity Questionnaire (IPAQ), validation of the tool (yes), last 3 months | Colorectal: 0.9 (0.69, 1.19) | Age, sex, country of birth, educational status, socioeconomic index, smoking status, alcohol intake, other physical activity domains, waist circumference |
| Simons et al. 2013  The Netherlands Cohort Study | 55-69 year, Cancer registry | Walking and cycling combined mode  duration (min/day) | **Men**  Colon: 0.94 (0.76, 1.17)  Proximal colon: 0.92 (0.68, 1.25)  Distal colon: 1.18 (0.88, 1.6)  Rectum: 1.1 (0.8, 1.52)  **Women**  Colon: 0.79 (0.6, 1.04)  Proximal colon: 0.85 (0.6, 1.19)  Distal colon: 0.75 (0.52, 1.1)  Rectum: 0.47 (0.27, 0.83) | Age, family history of colorectal cancer, smoking status, alcohol intake, BMI, meat intake, processed meat intake, and total energy intake. |
| Hou et al.  2004 | 30-74 years, Cancer registry | Walking and cycling separately  duration (min/day), lifetime | Walking  Colon (men): 0.71 (0.38, 1.06)  Colon (women): 0.57 (0.31, 0.93)  Cycling  Colon (men): 0.41 (0.21, 0.83)  Colon (women): 0.44 (0.12, 0.89) | Age, education, family income, marital status, total energy intake, intake of red meat, carotene, fibre, occupational physical activity, and leisure physical activity for both men and women and additional adjustment for number of pregnancies and menopausal status for women. |
| **Testicular** |  |  |  |  |
| Littman et al. 2009 | 18-44 years  Hospital records | Cycling commute in adolescence | 1.79 (0.91, 3.55) | Age, income, race, history of undescended testes, and total months of participation in competitive sports during grades 7–12 |
| Coldman et al. 1982 | Hospital records  (mean age or age range not provided) | Cycling commute in adolescence | 1 (0.7, 1.30) |  |
| **Prostate** |  |  |  |  |
| Zeegers et al 2005 | 55-69 years  Cancer registries | Walking and cycling combined mode  duration (min/day) | 0.85 (0.69, 1.05) | Age, alcohol intake from wine, BMI, energy intake, family history, level of education, gardening/doing odd jobs and sport/gymnastics |
| Hosseini et al. 2009 | Cancer registry  (mean age or age range not provided) | Walking only  Duration (hr/week) | 0.7 (0.4, 1.2) |  |
| **Ovarian** |  |  |  |  |
| Biesma et al. 2006 | 55-69 years  Cancer registry | Walking and cycling combined mode  Duration (min/day) | 0.83 (0.52, 1.31) | Age at baseline (years), height (cm), parity (number of children), age at first child (years), use of oral anticonceptives and BMI |
| **Liver, gallbladder and biliary tract** |  |  |  |  |
| Pang et al. 2021  China Kadoorie Biobank | 30-79 years, Surveillance system and national health insurance system | Commuting PA, MET h/day  Questions from EPIC and Shanghai Women Health Study, Validation of the tool (yes), previous year | Liver  Men: 0.85 (0.71, 1.01)  Women: 0.82 (0.6, 1.11)  Gallbladder and biliary tract cancer (GBTC)  Men: 0.92 (0.61, 1.37)  Women: 0.51 (0.28, 0.94) | Sex and region, and adjusted for age at baseline, education, household income, smoking, alcohol, self-rated health, diabetes, cardiovascular disease, respiratory disease, rheumatoid arthritis, and sedentary leisure time. |
| **Renal** |  |  |  |  |
| Xiao et al. 2014  Kidney Cancer Study | 20-79 year, Cancer surveillance, pathology reports | Walking and cycling combined mode, Duration (hr/wk) | Age of 20s  Black persons: 1.06 (0.73, 1.53)  White persons: 1.42 (1.10, 1.83)  Age of 50s  Black persons: 0.78 (0.50, 1.22)  White persons: 0.98 (0.67, 1.45) | Study center, age, sex, education, smoking status, and history of cancer among first-degree relatives. |
| **Lung** |  |  |  |  |
| Wong et al. 2022  UKB | 40-69 years, Cancer registries | Walk, cycling, public transport (reference mode: car) | Public transport, regular: 0.71 (0.37, 1.39)  Public transport, often: 1.58 (1.08, 2.33)  Walking, regular: 1.58 (0.87, 2.88)  Walking, often: 1.14 (0.67, 1.95)  Cycling, often: 1.14 (0.67, 1.95)  Walking and cycling, often: 1.1 (0.61, 1.99) | Average annual residential NO2, age, sex-smoking status, smoking duration, study site, race/ethnicity, body mass index, alcohol, income, and commute distance. |
| **Overall cancer incidence** |  |  |  |  |
| Patterson et al. 2020 | 16 year until retirement  Cancer registers | Walking, cycling, public transport (reference mode: car/motorcycle) | Walking: 0.93 (0.89, 0.97)  Cycling: 0.89 (0.82, 0.97)  Bus: 0.96 (0.91, 1.01)  Rail: 0.88 (0.83, 0.94) | Age, sex, housing tenure, marital status, ethnicity, university education, car access, population density, socioeconomic classification of occupation, Carstairs index quintile, long-term illness, and year entered study. |
| Panter et al. 2018  UKB | 40-69 years,  Cancer registries | Active patterns of commuting** (compared to car mode) | 1.00 (0.94, 1.08) | Age, sex, ethnicity, urban/rural, area-level deprivation, education, occupation, household income, cars owned, sleep time, screen time. fresh fruit, raw vegetables, cooked vegetables, smoking, PA at work, strenuous sport duration, other exercise duration, leisure walking duration, DIY duration, shift work, alcohol consumption, longstanding limiting illness/disability, BMI |
| Morales et al. 2017  UKB | 40-69 years, Cancer registries | Walking, cycling, mixed mode (walking), mixed mode (cycling)  (reference mode: non-active mode) | Walking: 0.93 (0.81, 1.07)  Cycling: 0.55 (0.44, 0.69)  Mixed mode (walking): 0.99 (0.88, 1.11)  mixed mode (cycling): 0.68 (0.57, 0.81) | Sex, age, deprivation index, ethnicity, smoking status, body mass index, leisure time, occupational and DIY physical activity, sedentary behaviour, and dietary intake prevalent chronic diseases at baseline (long- standing illness, diabetes, hypertension, CVD, cancer, and depression), in models that included participants with these conditions. |
| **Overall cancer mortality** |  |  |  |  |
| Patterson et al. 2020, UK | 16 year until retirement  Death registries | Walking, cycling, public transport (reference mode: car/motorcycle) | Walking: 0.98 (0.91, 1.06)  Cycling: 0.84 (0.73, 0.98)  Bus: 0.99 (0.9, 1.08)  Rail: 0.9 (0.8, 1.01) | Age, sex, housing tenure, marital status, ethnicity, university education, car access, population density, socioeconomic classification of occupation, Carstairs index quintile, long-term illness, and year entered study. |
| Panter et al. 2018  UKB | 40-69 years, Death registries | Active patterns of commuting** (compared to car mode) | 0.97 (0.84, 1.11) | Age (underlying timescale), sex, ethnicity, urban/rural, area-level deprivation, education, occupation, household income, cars owned, sleep time, screen time. fresh fruit, raw vegetables, cooked vegetables, smoking, PA at work, strenuous sport duration, other exercise duration, leisure walking duration, DIY duration, shift work, alcohol consumption, longstanding limiting illness/disability, BMI |
| Morales et al. 2017  UKB | 40-69 years, Death registries | Walking, cycling, mixed mode (walking), mixed mode (cycling)  (reference mode: non-active mode) | Walking: 1.1 (0.86, 1.42)  Cycling: 0.6 (0.4, 0.9)  Mixed mode (walking): 0.97, 0.77, 1.22  Mixed mode (cycling): 0.64 (0.45, 0.91) | Sex, age, deprivation index, ethnicity, smoking status, body mass index, leisure time, occupational and DIY physical activity, sedentary behaviour, and dietary intake prevalent chronic diseases at baseline (long- standing illness, diabetes, hypertension, CVD, cancer, and depression), in models that included participants with these conditions. |
| Sahlqvist et al. 2013 | 40-79 years  Death registries | Cycling only  Duration (min/wk) | 0.68 (0.28, 1.66) | Age, sex, education level and social class, smoking status, family history of cancer or cardiovascular disease, and all other physical activity. |
| Autenrieth et al. 2011 | 25-74 years  Population registries and death certificates | Transport PA  MONICA Optional Study on Physical Activity questionnaire, validation of the tool (yes) | 0.89 (0.64, 1.24) | Sex, BMI, systolic blood pressure, total-to-HDL cholesterol ratio, education, smoking status, alcohol consumption, myocardial infarction, stroke, diabetes, cancer, self-reported limited physical activity due to health problems, and other domains of physical activity. |
| Matthews et al. 2007 | 40-70 years  Death registries | Walking and cycling separately  MET h/day | Walking: 0.98 (0.75, 1.28)  Cycling: 0.55 (0.27, 1.11) | Age, marital status, education, household income, smoking, alcohol drinking, number of pregnancies; oral contraceptive use, menopausal status, other types of physical activity, chronic medical conditions |
| Batty et al. 2001 | 40-64 years  Death registries | Walking and cycling combined mode  duration (min/day) | Compared to the highest group  Men: 1 (0.9, 1.2) | Age, employment grade, body mass index, smoking, forced expiratory volume in 1 sec. |

^*^Comparison is between the highest and lowest levels of assessed mode/s if not specified with reference mode

**Any other patterns including walking, cycling, public transport, either alone or in combination with car

**Supplementary file 6: Newcastle-Ottawa Score of the studies (cohort studies)**

|  | **Selection** | | | | | **Comparability** | | **Outcome** | | | | |  |
| --- | --- | --- | --- | --- | --- | --- | --- | --- | --- | --- | --- | --- | --- |
| Study | **Representativeness of the exposed cohort**  (total or somewhat representative of the average population/ target group) in the community.) | **Selection of the non exposed cohort**  (drawn from the same community as the exposed cohort) | **Ascertainment of exposure**  secure record (eg surgical records) | **Ascertainment of exposure** (structured interview) | Demonstration that outcome of interest was not present at start of study | study controls for BMI  (height and weight, waist circumference, waist-hip ratio) | study controls for all other PA domains/ total PA | independent blind assessment | (record linkage) | Was follow-up long enough for outcomes to occur (=> 5 yr) | complete follow up - all subjects accounted for | subjects lost to follow up unlikely to introduce bias - small number lost (<= 10%) | **Total score** |
| Panter, 2018 | 1 | 1 |  |  | 1 | 1 |  |  | 1 | 1 |  |  | 6 |
| Pronk, 2011 | 1 | 1 |  | 1 | 1 |  |  |  | 1 | 1 |  | 1 | 7 |
| George, 2010 | 1 | 1 |  |  | 1 | 1 |  |  | 1 | 1 |  |  | 6 |
| Luoto, 2000 | 1 | 1 |  |  |  | 1 |  |  | 1 | 1 |  |  | 5 |
| Gierach, 2009 | 1 | 1 |  |  | 1 | 1 |  |  | 1 | 1 |  | 1 | 7 |
| Friberg, 2006 | 1 | 1 |  |  | 1 | 1 |  |  | 1 | 1 |  |  | 6 |
| Schouten, 2004 | 1 | 1 |  |  | 1 | 1 |  |  | 1 | 1 |  |  | 6 |
| Biesma, 2006 | 1 | 1 |  |  | 1 | 1 |  |  | 1 | 1 |  | 1 | 7 |
| Zeegers, 2005 | 1 | 1 |  |  | 1 | 1 |  |  | 1 | 1 |  | 1 | 7 |
| Wong, 2021 | 1 | 1 |  |  | 1 | 1 |  |  | 1 | 1 |  |  | 6 |
| Mahmood, 2018 | 1 | 1 |  | 1 | 1 |  |  |  | 1 | 1 |  |  | 6 |
| Simons, 2013 | 1 | 1 |  |  | 1 | 1 |  |  | 1 | 1 |  | 1 | 7 |
| Pang, 2020 | 1 | 1 |  | 1 | 1 |  |  |  | 1 | 1 |  | 1 | 7 |
| Patterson, 2021 | 1 | 1 |  | 1 | 1 |  |  |  | 1 | 1 |  |  | 6 |
| Celis-Morales, 2017 | 1 | 1 |  |  | 1 | 1 |  |  | 1 |  |  |  | 5 |
| Matthews, 2007 | 1 | 1 |  | 1 | 1 |  |  |  | 1 | 1 |  | 1 | 7 |
| Sahlqvist, 2013 | 1 | 1 |  | 1 | 1 |  |  |  | 1 | 1 |  |  | 6 |
| Batty, 2001 | 1 | 1 |  |  | 1 | 1 |  |  | 1 | 1 |  | 1 | 7 |
| Autenrieth, 2011 | 1 | 1 |  | 1 | 1 | 1 | 1 |  | 1 | 1 |  | 1 | 9 |

**Supplementary file 7: Newcastle-Ottawa Score of the studies (case control studies)**

|  | **Selection** | | | | **Comparability** | | **Exposure** | | | | **Total score** |
| --- | --- | --- | --- | --- | --- | --- | --- | --- | --- | --- | --- |
|  | Adequacy of case definition | Consecutive or obviously representative series of cases | Community controls | No history of disease (endpoint) | Study controls for BMI (height and weigth, wasit circumference, waist-hip ratio) | study controls for all other PA domains/ total PA | secure record (eg surgical records) | Structured interview where blind to case/control status | Same method of ascertainment for cases and control | Same response rate for both groups |  |
| Gomes, 2022 | 1 | 1 | 1 | 1 |  |  |  |  | 1 |  | 5 |
| Azubuike, 2022 | 1 | 1 |  | 1 | 1 |  |  |  | 1 |  | 5 |
| Si, 2015 | 1 | 1 | 1 | 1 | 1 |  |  |  | 1 |  | 6 |
| Mathew, 2009 | 1 | 1 |  | 1 | 1 |  |  |  | 1 | 1 | 6 |
| Steindorf, 2003 | 1 | 1 | 1 |  | 1 | 1 |  |  | 1 |  | 6 |
| John, 2003 | 1 | 1 | 1 | 1 | 1 | 1 |  |  | 1 |  | 7 |
| Matthews, 2001 | 1 | 1 | 1 | 1 |  |  |  |  | 1 |  | 5 |
| Marcus, 1999 | 1 | 1 | 1 | 1 |  |  |  |  | 1 |  | 5 |
| John, 2010 | 1 | 1 | 1 | 1 | 1 |  |  |  | 1 |  | 6 |
| Matthews, 2005 | 1 | 1 | 1 | 1 | 1 | 1 |  |  | 1 |  | 7 |
| Hosseini, 2009 | 1 | 1 | 1 |  |  |  |  |  | 1 |  | 4 |
| Coldman, 1982 | 1 | 1 |  | 1 | 1* | 1** |  |  | 1 |  | 6 |
| Littman, 2009 | 1 | 1 | 1 |  | 1* | 1** |  |  | 1 |  | 6 |
| Hou, 2004 | 1 | 1 | 1 |  | 1 | 1 |  |  | 1 |  | 6 |
| Xiao, 2014 | 1 | 1 | 1 | 1 |  |  |  |  | 1 |  | 5 |

*control undescended testes, **control other riding activities

**Supplementary file 8: Risks estimates used in the meta-analyses**

**(provided in the separate excel sheet)**

## **Supplementary file 9: Forest plots**

1. **Highest versus lowest group***

### **Breast cancer**


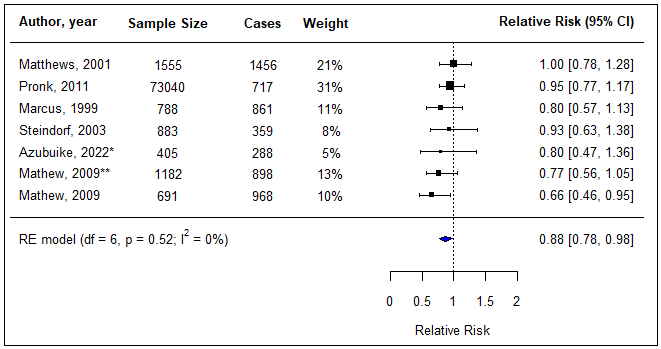


**Figure 1: Forest plot of breast cancer and walking (highest vs lowest group)**

RE= Random Effect Model, * premenopausal, ** postmenopausal


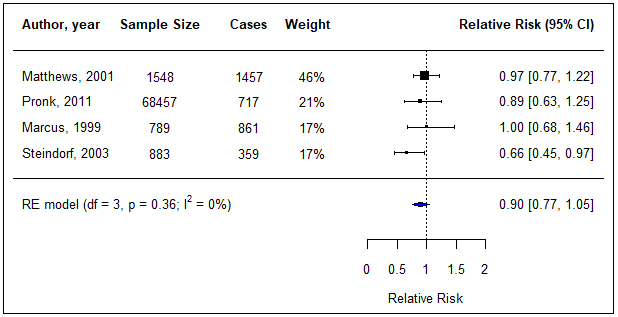


**Figure 2: Forest plot of breast cancer and cycling (highest vs lowest group)**

RE= Random Effect Model

*Confidence interval of some relative risks shown in forest plots are slightly different from original values due to the transformation of the log scale (used in meta-analysis) to the original scale when it is shown in a forest plot.


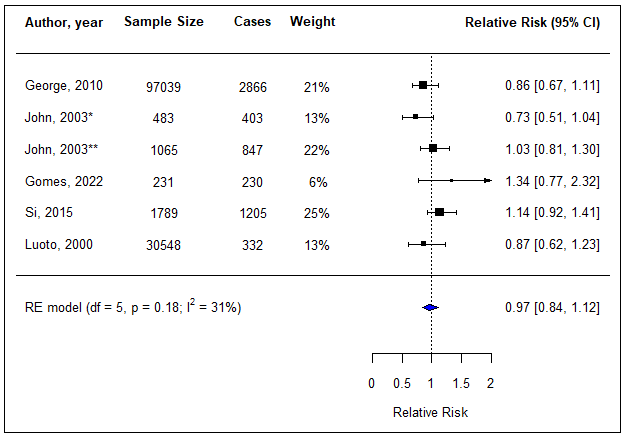


**Figure 3: Forest plot of breast cancer and walking and cycling combined mode (highest vs lowest group)**

RE= Random Effect Model, * premenopausal, ** postmenopausal

**Endometrial cancer**


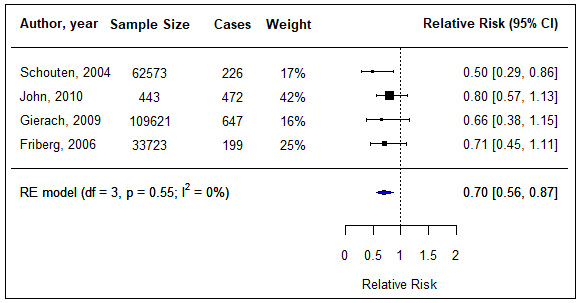


**Figure 4: Forest plot of meta-analysis endometrial cancer and walking and cycling combined mode (highest vs lowest group)**

RE= Random Effect Model

### **Colorectal cancer**


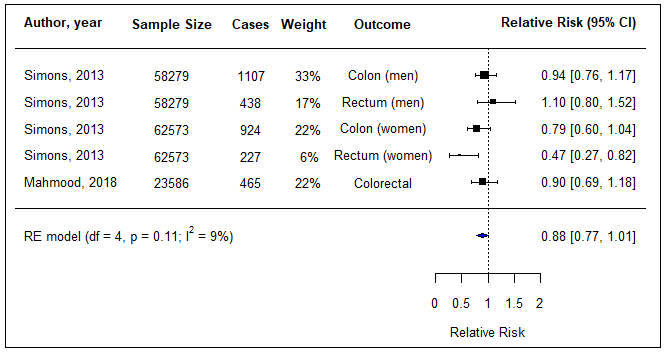


**Figure 5: Forest plot of meta-analysis of colorectal cancer and walking and cycling combined mode (highest vs lowest group)**

RE= Random Effect Model

### **Testicular cancer**


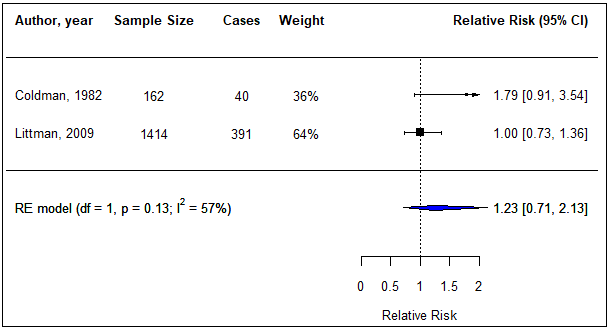


**Figure 6: Forest plot of testicular cancer and cycle commuting in adolescence (highest vs lowest group)**

RE= Random Effect Model

**Overall cancer mortality**


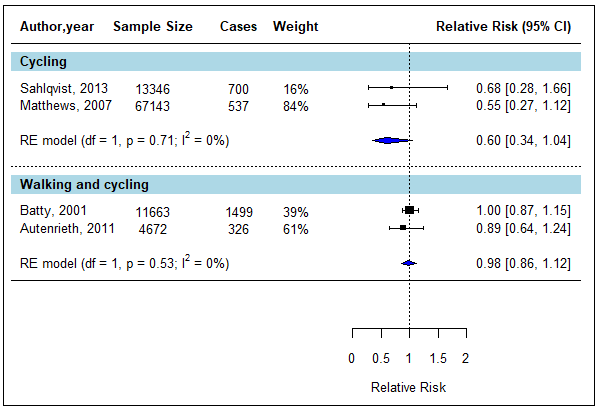


**Figure 7: Forest plot of meta-analysis of overall mortality and cycling only, and walking and cycling combined mode (highest vs lowest group)**

RE= Random Effect Model

1. **10 MET hour increment per week**

**Breast cancer**

**
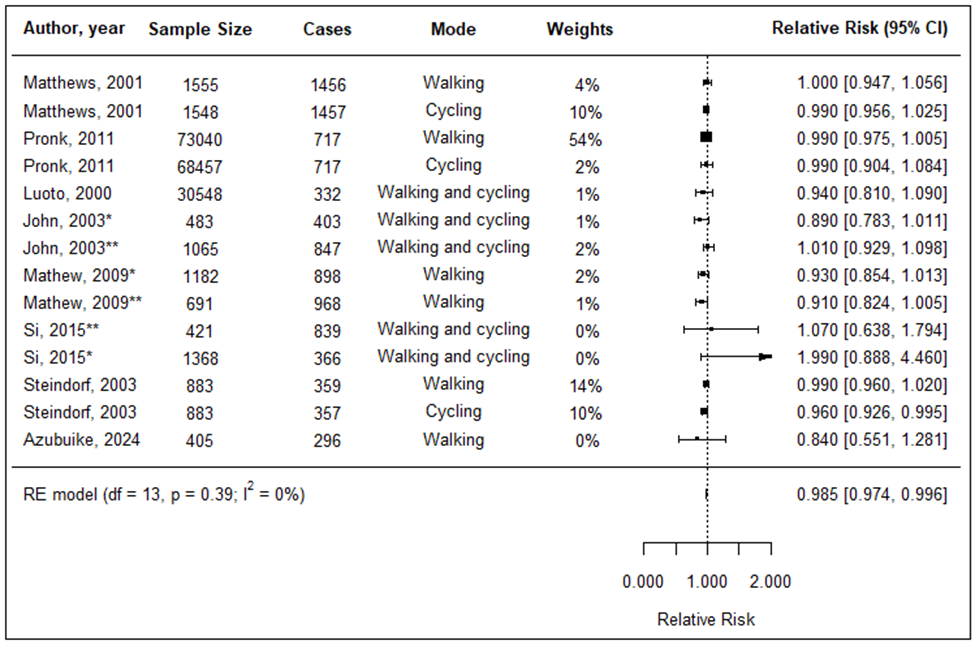
**

**Figure 8: Forest plot of breast cancer and 10 MET hour increment per week**RE= Random Effect Model; Note: three decimal places are presented to show the upper limit of the result

**Endometrial cancer**


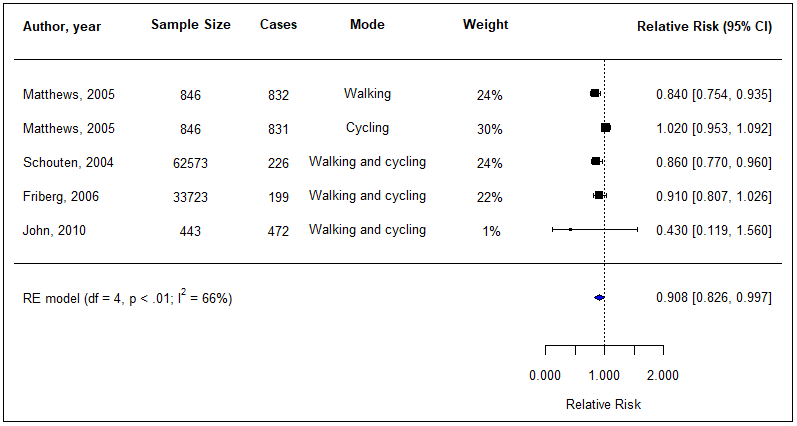


**Figure 9: Forest plot of endometrial cancer and 10 MET hour increment per week**

RE= Random Effect Model

**Colorectal cancer**


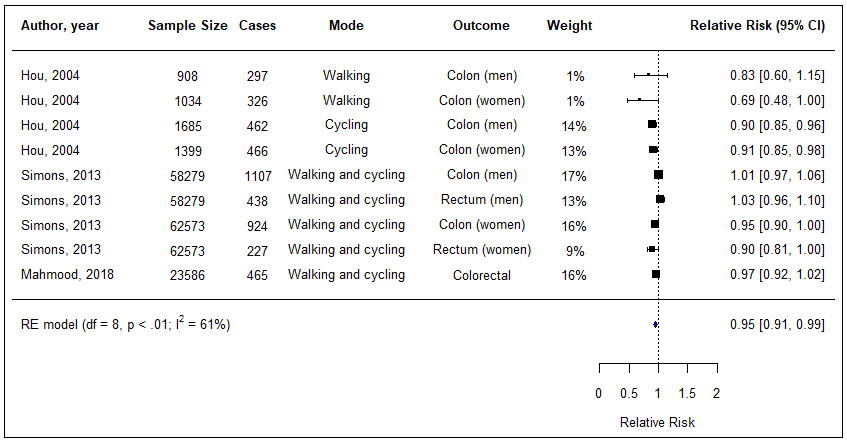


**Figure 10:Forest plot of colorectal cancer and 10 MET hour increment per week**

RE= Random Effect Model

**Prostate cancer and overall cancer mortality**


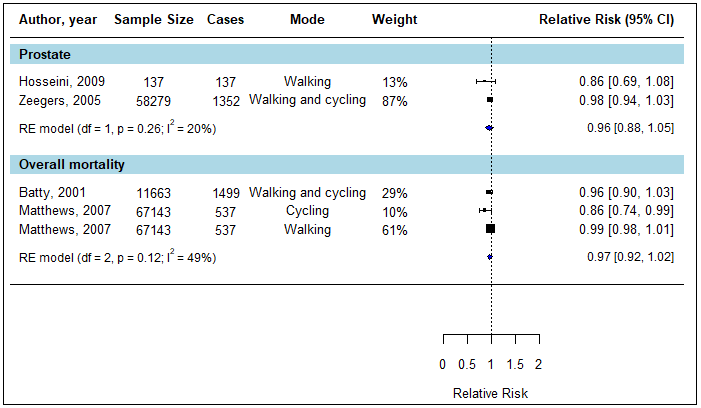


**Figure 11:Forest plot of prostate cancer, overall cancer mortality and 10 MET hour increment per** **week**

RE= Random Effect Model

**Supplementary file 10: Sub-group analyses and covariates adjustment**


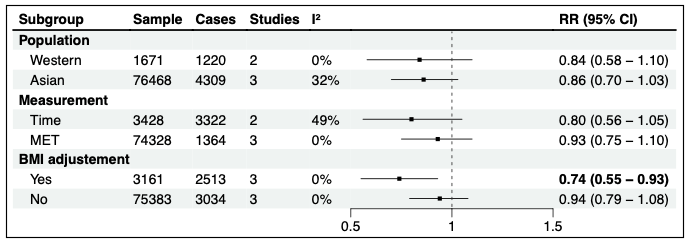


**Figure 12: Sub-group analysis for breast cancer and walking (highest vs lowest group)**

MET = Metabolic Equivalent Task, BMI = Body Mass Index, RE = random effects model, I^2= I^2^ statistics


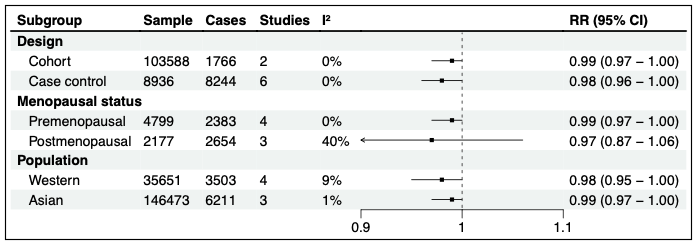


**Figure 13: Sub-group analysis for breast cancer and 10 MET hour increment per week**


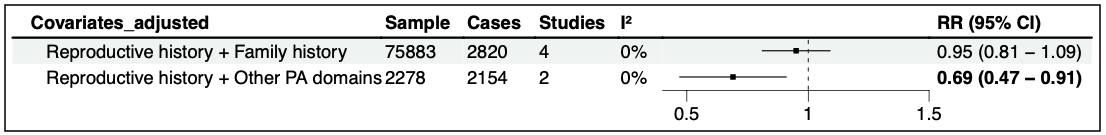


**Figure 14: Results by covariates adjustment (walking, highest vs lowest group)**

**Supplementary file 11: Sensitivity analyses**

Sensitivity analyses were conducted by removing one risk estimate at a time from the meta-analyses.


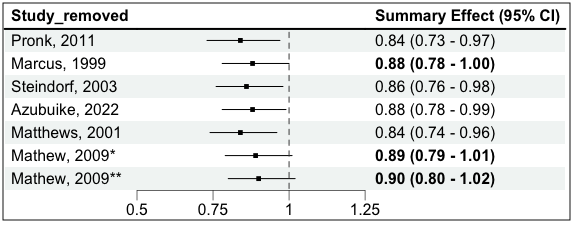


**Figure 15: Sensitivity analysis of breast cancer and walking (highest vs lowest group)**

*premenopausal, **postmenopausal


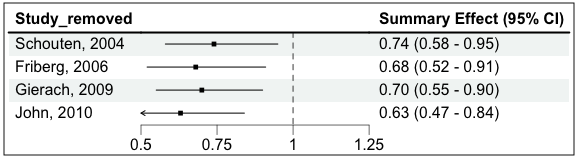


**Figure 16: Sensitivity analysis of endometrial cancer and walking and cycling combined mode**

(highest vs lowest group)

**Supplementary files 12: Funnel plots**


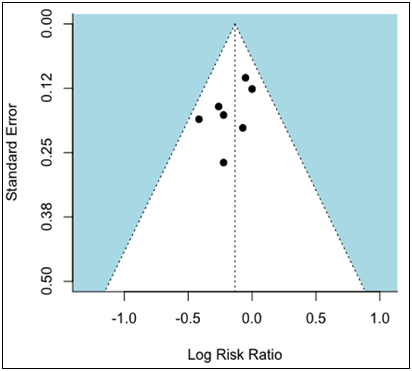


**Figure 17: Funnel plot for breast cancer and walking**

Egger's regression test p= 0.17, Begg's ranks correlation test p = 0.56

**
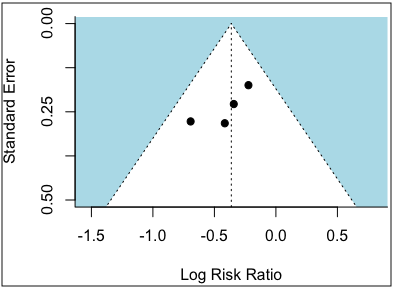
**

**Figure 18: Funnel plots for endometrial cancer and walking and cycling combined mode**

Egger's regression test p= 0.22, Begg's ranks correlation test p = 0.33

1. Compedium of Physical Activities. Accessed August 3, 2023. https://sites.google.com/site/compendiumofphysicalactivities/Activity-Categories/walking?authuser=0 [↑](#footnote-ref-1)
